# Predictive and Prognostic Relevance of ABC Transporters for Resistance to Anthracycline Derivatives

**DOI:** 10.3390/biom15070971

**Published:** 2025-07-06

**Authors:** Rümeysa Yücer, Rossana Piccinno, Ednah Ooko, Mona Dawood, Gerhard Bringmann, Thomas Efferth

**Affiliations:** 1 Department of Pharmaceutical Biology, Institute of Pharmaceutical and Biomedical Sciences, Johannes Gutenberg University, Staudinger Weg 5, 55128 Mainz, Germany; ryuecer@students.uni-mainz.de (R.Y.); modawood@uni-mainz.de (M.D.); 2 Institute of Molecular Biology, Ackermannweg 4, 55128 Mainz, Germany; r.piccinno@imb-mainz.de; 3 Laboratory of Cell Biology, Center for Cancer Research, National Cancer Institute, National Institutes of Health, Bethesda, MD 20892, USA; ookoea@nih.gov; 4 Department of Biological Sciences, School of Natural and Applied Sciences, Masinde Muliro University of Science and Technology, Kakamega 190-50100, Kenya; 5 Department of Molecular Biology, Faculty of Medical Laboratory Sciences, Al-Neelain University, Khartoum 12702, Sudan; 6 Institute of Organic Chemistry, University of Würzburg, Am Hubland, 97074 Würzburg, Germany; gerhard.bringmann@uni-wuerzburg.de

**Keywords:** anthracyclines, chemotherapy, drug development, Kaplan–Meier statistics, molecular docking, multidrug resistance, the cancer genome atlas (TCGA)

## Abstract

Anthracyclines have been clinically well established in cancer chemotherapy for decades. The main limitations of this drug class are the development of resistance and severe side effects. In the present investigation, we analyzed 30 anthracyclines in a panel of 59 cell lines of the National Cancer Institute, USA. The log_10_IC_50_ values varied from −10.49 M (3′-deamino-3′-(4″-(3″-cyano)morpholinyl)-doxorubicin, **1**) to −4.93 M (*N*,*N*-dibenzyldaunorubicin hydrochloride, **30**). Multidrug-resistant NCI-ADR-Res ovarian cancer cells revealed a high degree of resistance to established anthracyclines (between 18-fold to idarubicin (**4**) and 166-fold to doxorubicin (**13**) compared to parental, drug-sensitive OVCAR8 cells). The resistant cells displayed only low degrees of resistance (1- to 5-fold) to four other anthracyclines (**7**, **18**, **28**, **30**) and were even hypersensitive (collaterally sensitive) to two compounds (**1**, **26**). Live cell time-lapse microscopy proved the cross-resistance of the three chosen anthracyclines (**4**, **7**, **9**) on sensitive CCRF/CEM and multidrug-resistant CEM/ADR5000 cells. Structure–activity relationships showed that the presence of tertiary amino functions is helpful in avoiding resistance, while primary amines rather increased resistance development. An α-aminonitrile function as in compound **1** was favorable. Investigating the mRNA expression of 49 ATP-binding cassette (ABC) transporter genes showed that *ABCB1/MDR1* encoding P-glycoprotein was the most important one for acquired and inherent resistance to anthracyclines. Molecular docking demonstrated that all anthracyclines bound to the same binding domain at the inner efflux channel side of P-glycoprotein with high binding affinities. Kaplan–Meier statistics of RNA sequencing data of more than 8000 tumor biopsies of TCGA database revealed that out of 23 tumor entities high *ABCB1* expression was significantly correlated with worse survival times for acute myeloid leukemia, multiple myeloma, and hepatocellular carcinoma patients. This indicates that *ABCB1* may serve as a prognostic marker in anthracycline-based chemotherapy regimens in these tumor types and a target for the development of novel anthracycline derivatives.

## 1. Introduction

Although chemotherapy of malignant diseases is one of the mainstays in clinical oncology, the activity of anticancer drugs is frequently hampered by the development of resistance. Even worse, cancer cells might not only be unresponsive to single drugs but also to multiple drugs at the same time, independently of whether or not the tumors have been in contact with these drugs. Cross-resistance phenomena had already been recognized in the 1970s prior to description of the classical multidrug-resistance phenotype [1,2].

A defined profile of cross-resistance between anthracyclines, *Vinca* alkaloids, taxanes, epipodophyllotoxins, and some antibiotics has been described, named multidrug resistance. The molecular mode of action was an efflux transporter termed P-glycoprotein encoded by the *ABCB1/MDR1* gene [3,4,5].

Apart from this “classical” MDR phenotype, other cross-resistance phenomena were discovered that were not conferred by P-glycoprotein and the *ABCB1/MDR1* gene. Atypical, cross-resistance to partwise other drugs or drug classes were mediated by DNA topoisomerases I and II [6,7] and other efflux transporters belonging to the same gene family as the *ABCB1/MDR1* gene, such as *ABCC1/MRP1* and *ABCG2/BCRP* [8,9]. With the advent of more sophisticated molecular biological methods and cloning techniques, it became apparent that these efflux transporters belong to the family of ATP-binding cassette (ABC) transporters. The human genome contains 49 ABC transporters expressed in diverse healthy organs and tissues fulfilling numerous physiological functions in the body [10,11,12]. ABC transporters have been identified throughout the different kingdoms of living organisms, e.g., bacteria, fungi, animals, and even in plants [13,14,15,16,17]. Twelve out of the 49 human ABC transporters have been associated with drug resistance phenomena in tumors [18].

Therefore, the question arises whether it might be a better strategy to search for novel non-cross-resistant drugs rather than for ABC transporter inhibitors. Given the generally strong anticancer activity of anthracyclines, it might be attractive to identify anthracycline derivatives that do not belong to the MDR phenotype. Anthracyclines are a main class of drugs involved in several cross-resistance profiles mediated by different ABC transporters [18]. Natural products derived from *Streptomyces* strains (doxorubicin, daunorubicin) and semisynthetic derivatives (epirubicin, idarubicin) are clinically well established and applied for a wide variety of hematological and solid malignancies (Minotti et al., 2004 [19]). Anthracyclines belong to the class of anticancer drugs that serve as substrates (better termed “translocates” because there is no substrate-specific enzymatic reaction happening during the transport process) [20].

Efforts have been undertaken to develop novel anthracycline analogs with improved features, including less cross-resistance of ABC-transporter-expressing tumors (Scudder et al., 1988 [21]). However, a systematic analysis with comparable data of a larger number of anthracyclines in a uniform dataset of a large panel of tumor cell lines is still missing. The repository of the Developmental Therapeutics Program of the National Cancer Institute (Bethesda, MD, USA) may offer the opportunity for such a systematic analysis [22].

A prediction to establish this concept is that ABC transporters really influence the survival of cancer patients. Indeed, there are also many reports on the prognostic relevance of ABC transporters for the survival probability of cancer patients. Despite many reports demonstrating that high ABC transporter expression is associated with short survival times, there are also data not supporting this viewpoint [23,24,25]. Finally, the real clinical relevance has not been unambiguously clarified. Therefore, it is important not only to identify anthracyclines that are not transported by ABC transporters but also to clarify which ABC transporters may impede the action of which anthracycline in a clinical setting. In this context, it is important to know which ABC transporter predicts the survival time of patients. The Cancer Genome Atlas (TCGA) project offers a unique opportunity to investigate the prognostic relevance of a huge number of genes, including ABC transporters [26]. The advance is that the gene expression has been determined in a non-directed manner, i.e., not with the aim of demonstrating the value of a certain gene or gene family (i.e., the ABC transporter family). Therefore, correlations of TCGA-based gene expression data with the patients’ survival time may be without bias, providing reliable data.

Therefore, we studied a total of 30 different anthracycline derivatives in the NCI60 cell line panel of the National Cancer Institute (USA) and correlated their cellular response with the expression of 49 ABC transporters in these tumor cell lines. Another aim of our investigations was to study the prognostic relevance of P-glycoprotein/*ABCB1/MDR1* as a main mechanism for resistance to anthracyclines for the survival time of cancer patients using Kaplan–Meier statistics, evaluating data from the TCGA database. Combining both approaches revealed that non-cross-resistant anthracyclines could be identified that might be promising for the treatment of leukemia and multiple myeloma, where *ABCB1/MDR1* was a significantly worse prognostic factor.

## 2. Materials and Methods

### 2.1. Cell Lines

The cell lines of the NCI-60 drug screening panel are documented in the repository of the Developmental Therapeutics Program of the National Cancer Institute (NCI, Bethesda, MD, USA). Their genetic and molecular characterization is available on the NCI website and documented in related studies [27,28,29].

Cell lines with inherent drug resistance: The panel of cell lines with various degrees of responsiveness to anticancer drugs and without prior drug selection were derived from leukemia (CCRF-CEM, HL-60(TB), K-562, MOLT-4, RPMI-8226, SR), melanoma (LOXIMVI, MALME-3M, M14, MDA-MB-435, SK-MEL-2, SK-MEL-28, SK-MEL-5, UACC-257, UACC-62), brain tumors (SF-268, SF-295, SF-539, SNB-19, SNB-75, U251), and from carcinomas of the lung (A549/ATCC, EKVX, HOP-62, HOP-92, NCI-H226, NCI-H23, NCI-H322M, NCI-H460, NCI-H522), colon (COLO205, HCC-2998, HCT-116, HCT-15, HT29, KM12, SW-620), ovary (IGROV1, OVCAR-3, OVCAR-4, OVCAR-5, OVCAR-8, SK-OV-3), kidney (786-0, A498, ACHN, CAKI-1, RXF393, SN12C, TK-10, UO-31), prostate (PC-3, DU-145), and breast (MCF7, MDA-MB-231/ATCC, HS578T, BT-549, T-47D). These cell lines were not priorly exposed to anticancer drugs and were used to investigate their inherent sensitivity and resistance to anthracycline derivatives. The MDA-N melanoma cell line of the NCI tumor panel, which is also frequently used for drug testing, has been omitted from the present analysis. This cell line has been generated by transfecting MDA-MB-435 melanoma cells with the *HER2/neu* gene (encoding the human epidermal growth factor receptor 2). We deemed it unsuitable for investigating inherent drug resistance because it was genetically modified. NCI-ADR-Res cells have been selected for doxorubicin resistance from OVCAR-8 wild-type cells [30]. The multidrug-resistance phenotype and P-glycoprotein expression of NCI/ADR-Res cell line has been described previously [31,32].

### 2.2. Drugs

**Figure 1:**
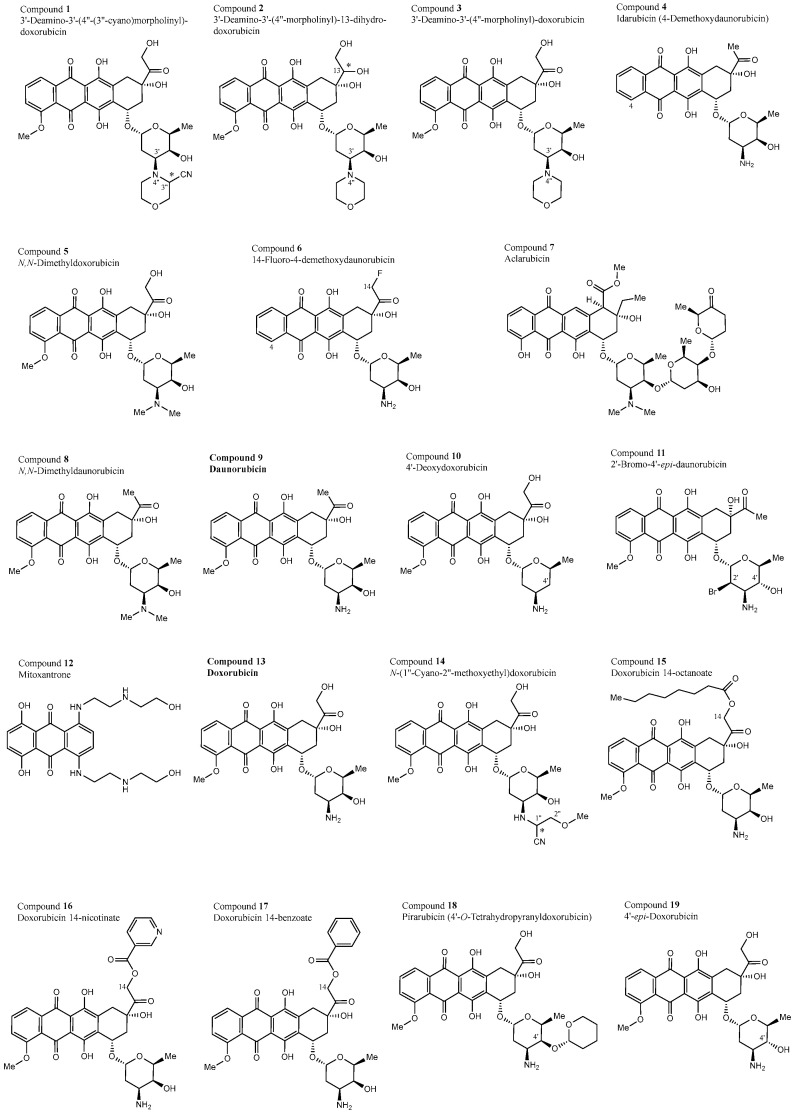

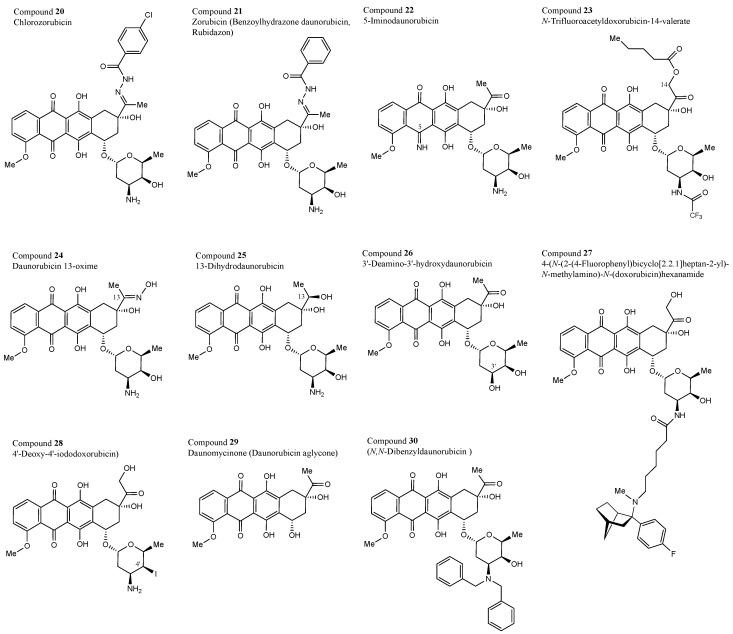
Two-dimensional chemical structures of the 30 anthracyclines investigated. Parent compounds are shown in bold. * Configuration at this stereogenic center not indicated in the literature. Therefore, both possible diastereomers were included in the molecular docking.

The NCI repository contains more than 200,000 synthetic and natural compounds that have been tested for their cytotoxicity toward the above-mentioned panel of NCI tumor cell lines by using the sulforhodamine B assay [33]. The 50% inhibition concentrations (IC_50_) were calculated from the corresponding dose–response curves (https://dtp.cancer.gov/discovery_development/nci-60/methodology_HTS384.htm, accessed on 1 November 2024) [34]. We selected 30 anthracycline derivatives (Table 1, Figure 1) and three doxorubicin complexes linked with transferrin, hydroxyethyl starch, or DNA fragments from the NCI database. To search the database, we screened it for chemical names containing the suffixes “-rubicin” and “-mycin”, and then inspected the identified compounds for their chemical structures to determine whether they contained the anthracycline scaffold (https://dtp.cancer.gov/dtpstandard/cancerscreeningdata/index.jsp, accessed on 1 November 2024).

### 2.3. Expression of ABC Transporter Genes

The mRNA expression of ABC transporter genes as measured by qRT-PCR and microarray hybridization has been reported and deposited in the NCI database [35] (https://dtp.cancer.gov/, accessed on 1 November 2024). The mRNA expression of these genes has been exemplarily validated by two different methods (qRT-PCR and microarray hybridization). For microarray hybridization, two different array systems have been used (Affimetrix U95Av2A-E and U133A/B). Furthermore, the expression of micro-RNAs (miR) and miR precursors has been analyzed by qRT-PCR. MiR-451, miR-027a prec, and miR-27aN are known to regulate essential functions in cancer cells such as proliferation, differentiation, and cell death [36]. Due to their involvement in carcinogenesis and cancer progression, they were also termed “onco-miRs”. All three onco-miRs regulate the expression of *ABCB1/MDR1* [37,38,39].

### 2.4. Molecular Docking

The pdb file of P-glycoprotein (*ABCB1*-encoded protein) (pdb: 8y6h) was downloaded from the RCSB Protein Data Bank (www.rcsb.org/structure/8Y6H, accessed on 1 November 2024). The protein was modified on AutoDock 1.5.6 (https://ccsb.scripps.edu/mgltools/1-5-6/, accessed on 1 November 2024) by adding polar hydrogens, repairing missing atoms, and adding Kollman charges. The binding site was determined as the drug binding site, and the coordinates were set for the grid center at x: 150.951, y: 149.316, z: 149.745, with a spacing of 0.5 and grid dimensions of 50 × 40 × 70 points along the x, y, and z axes, respectively. The Lamarckian algorithm was applied with 250 runs and 2,500,000 energy evaluations. The parent compounds (doxorubicin and daunorubicin), well-identified compounds (aclarubicin and zorubicin), and elacridar as positive control were downloaded as “sdf” files from the ZINC database (https://zinc.docking.org/substances/, accessed on 1 November 2024). The rest of the compounds were generated by modification of these compounds. “Sdf” files were converted to “pdb” files by Chem3D (https://revvitysignals.com/products/research/chemdraw, accessed on 1 November 2024) with energy minimization and converted to “pdbqt” files by PyRx (https://pyrx.sourceforge.io/, accessed on 1 November 2024). Molecular docking was conducted using AutoDock 4.2 (https://autodock.scripps.edu/download-autodock4/, accessed on 1 November 2024) and the supercomputer MOGON of Johannes Gutenberg University Mainz (https://hpc.uni-mainz.de/, accessed on 1 November 2024), which is a member of the AHRP (Alliance for High-Performance Computing in Rhineland–Palatinate, ahrp.info). Lowest binding energies (LBEs), with their predicted inhibition constants (pK_i_ values), were obtained from AutoDock-created dlg files. The conformations corresponding to the LBEs were visualized using Discovery Studio Visualizer (https://discover.3ds.com/discovery-studio-visualizer-download, accessed on 1 November 2024), and eight interacting amino acids were noted from 2D diagrams of each compound.

### 2.5. Hierarchical Cluster Analysis and Statistical Methods

Cluster models are well established for gene expression profiling in the molecular pharmacology of cancer [40,41]. The interval-scaled linear correlations with significance values (*p*) and rank correlation coefficients (r) between the log_10_IC_50_ values for 30 anthracyclines and the mRNA expression values for 49 ABC transporter genes were first calculated by Pearson’s correlation test implemented in WinSTAT (Kalmia, Cambridge, MA, USA).

Hierarchical cluster analyses were performed using the Ward method implemented in the WinSTAT program (Kalmia, students version, Cambridge, MA, USA). The program automatically omitted missing values and standardized the variables by transforming the data into values with mean = 0 and variance = 1. The correlation rank coefficients were used for hierarchical cluster calculation. All values of the matrix were assembled in cluster trees (dendrograms), which were used to generate a two-dimensional cluster image map.

The distribution of anthracycline derivatives or tumor cell lines in different branches of the dendrograms was calculated by the χ^2^-test (WinSTAT, Kalmia, Cambridge, MA, USA).

### 2.6. Resazurin Cell Viability Assay

The resazurin reduction assay was conducted to measure the inhibition of drug-sensitive CCRF-CEM cells and their multidrug-resistant, P-glycoprotein (ABCB1) overexpressing subline CEM/ADR 5000, which were obtained from Prof. Axel Sauerbrey (University of Jena, Jena, Germany). The cell lines were treated with doxorubicin, daunorubicin, and idarubicin, which were provided by the University Medical Center of the Johannes Gutenberg University (Mainz, Germany), and with aclarubicin (HY-N2306) and pirarubicin (HY-13725), which were supplied by MedChemExpress, Monmouth Junction, NJ, USA). The assay was performed as previously described [42].

### 2.7. Live Cell Time-Lapse Microscopy

Drug-sensitive CCRF-CEM and resistant CEM/ADR5000 cells (40,000 cells) were seeded in 96-well plates and treated with compounds **9**, **4**, or **7** (daunorubicin, idarubicin, or aclarubicin) at a final concentration of 5 µM. To monitor differences in compound uptake dynamics, time-lapse live cells microscopy was performed using the Incucyte^®^ SX5 Live-Cell Analysis System (Sartorius, Göttingen, Germany) equipped with an optical module for phase contrast/green/orange/NIR channels. Phase contrast images and “orange” channel (excitation filter 546–568 nm; emission filter 576–639 nm; 400 ms exposure) of non-adherent CCRF-CEM and CEM/ADR5000 cells were acquired via a dry 20x/NA 0.45 (0.62 µm/pixel) lens. Immediately after compounds were added to the culture media, cellular uptake was monitored every 3 min for the first 0.5 h, then every 15 min for a total duration of 3 h. Incucyte^®^ image analysis was performed via a built-in deep learning software module for non-adherent cell segmentation. Briefly, label-free cell detection was achieved by setting the diameter of objects to 15 µm and optimal masking was achieved by setting threshold and texture sensitivity to 5. Background-subtracted images were automatically calculated based on a best-fit polynomial algorithm, and fluorescence signal within the masked cells was automatically analyzed. Average object mean intensity of fluorescence signal was calculated and results exported as text files. Plotting of average object mean intensity was performed via GraphPad Prism 10.1.2.

### 2.8. Kaplan–Meier Survival Statistics

Kaplan–Meier statistics are well known in clinical oncology for calculating the survival probability of patients according to their clinical, biochemical, or molecular parameters. In the present study, we used the KM Plotter algorithm (https://kmplot.com/analysis/, accessed on 1 November 2024) as described [43,44]. The pan-cancer database of the KM Plotter consists of RNA sequencing data from 7489 biopsies of 23 different tumor types from TCGA. In addition, we used the gene chip-based mRNA expression repository of 1608 acute myeloid leukemia and 1416 multiple myeloma samples.

## 3. Results

### 3.1. Structural Properties of the 30 Test Compounds

The structures of the 30 anthracyclines investigated show a close structural similarity to the natural precedent, daunorubicin (compound **9**), and all form a relatively narrow structural family (Figure 1). They all (except for compound **12**) possess a linear tetracyclic ring system, consisting of a bis-hydroxylated tricyclic anthraquinone (as a DNA-intercalating chromophore), complemented by a fourth, partially saturated ring, which is linearly annulated on the “right” side, and all (except for compounds **12** and **29**, and compound **26**, which has a deoxy sugar moiety, instead) are equipped with a characteristic amino sugar, jointly forming the minor groove-binding moiety and enzyme-interacting domain. Consequently, all 30 compounds have the following in common:

• The presence of an amino sugar entity (even with two further yet nitrogen-free sugar units in the case of **7**), but none for **12**, **26**, and **29** (see above)
• Two phenolic OH groups in the anthraquinone part (invariably, without any exception)
• In most cases two further aliphatic OH groups (three for the nine structures **1**, **3**, **5**, **13**, **14**, **19**, **25**, **26**, and **27**, and even four in the case of compound **2**, but in no case just one OH group or even none)
• Except for compound **23**, whose nitrogen is amidated (and there is another, additional amide nitrogen in **27**) and compound **26**, all contain basic amino functions, in most cases primary ones (-NH_2_); only in two cases (**2**, **12**) is it a secondary one (-NHR); in **12**, there are even four secondary amino groups; and in eight cases (**1**, **2**, **3**, **5**, **7**, **8**, **27**, and **30**) they are tertiary amines (-NR_2_).

Less frequently occurring are halogen substituents:

• fluorine: in compounds **6**, **23** (CF_3_ group), and **27**
• chlorine: **20**
• bromine: **11**
• iodine: **28**.

### 3.2. Stereochemistry

**Figure 2:**
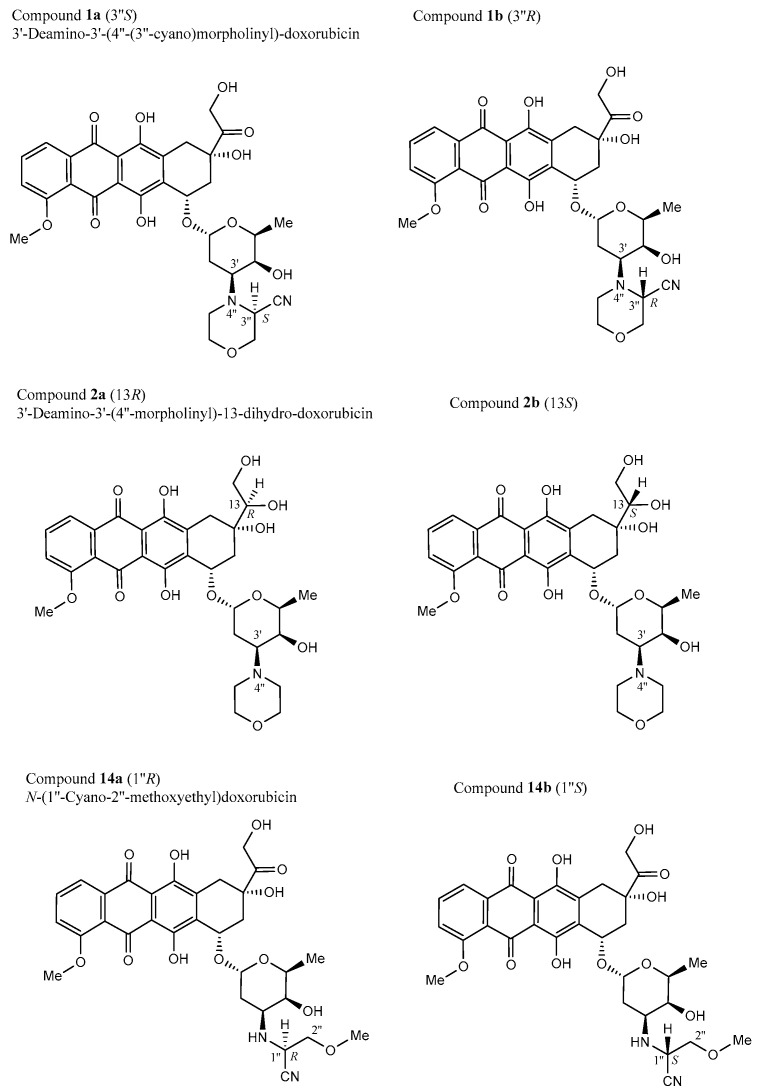
Epimeric structures of the two respective diastereomers of compounds **1**, **2**, and **14**. We arbitrarily named the stronger-binding diastereomers of **1**, **2**, and **14** as “**a**” (like “**1a**”) and the weaker-binding one as “**b**”.

All compounds investigated in this study are chiral anthracyclines—linearly annulated tetracyclic molecules, which owe their chirality to a chiral side chain in the ‘northeastern’ part of the molecule and/or to a chiral sugar unit in the ‘southwest’, in some cases complemented by further chiral subunits on the sugar part. An exception is compound **12** (mitoxantrone), an achiral tricyclic anthraquinone devoid of any stereogenic centers and with a constitutionally symmetric structure. All other compounds are chiral, possessing between five (compound **10**) and 13 (compound **7**) stereocenters, all stereochemically defined and indicated—except for three compounds, **1**, **2**, and **14**. In each of these cases, one center of chirality was configurationally unknown (at C-3″ for **1**, at C-13 for **2**, and at C-1″ for **14**), evidently resulting from their semi-synthetic preparation. Given the impact of chirality on the bioactivity of chiral agents, we thus investigated, in all these three cases, both of the two possible diastereomers, **1a**/**1b**, **2a**/**2b**, and **14a**/**14b** (as defined in Figure 2) separately when performing molecular docking investigations.

### 3.3. Structure–Activity Relationships Regarding the Cytotoxicity of the Anthracyclines

**Figure 3:**
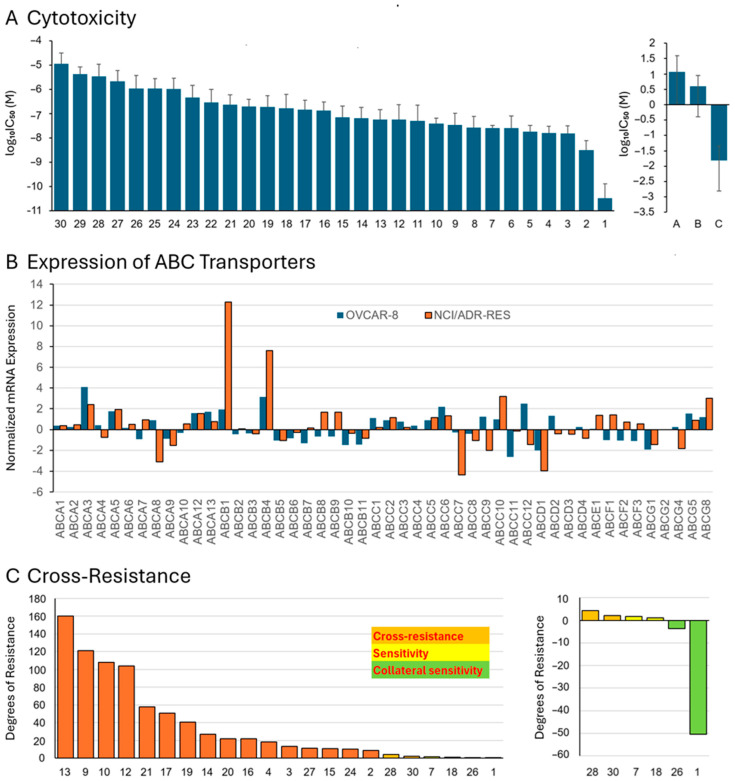
Cytotoxicity and cross-resistance of 30 anthracyclines in the NCI tumor cell line panel. (**A**) Cytotoxicity as measured by the sulforhodamine B assay in 58 cell lines derived from leukemia, melanoma, brain tumors, as well as carcinomas of the colon, lung, ovary, kidney, prostate, and breast. (**B**) Expression of 49 ABC transporter genes in parental drug-sensitive OVCAR-8 and multidrug-resistant NCI-ADR Res ovarian carcinoma cells by qRT-PCR. The multidrug-resistance phenotype of NCI/ADR-Res cell line has been earlier described [31,32]. (**C**) Cross-resistance of 22 anthracyclines in multidrug-resistant NCI-ADR-Res cells. The degrees of resistance have been determined by dividing the IC_50_ value for the corresponding drug of NCI-ADR Res cells by the one of OVCAR-8 cells.

The 30 anthracyclines have been tested for their cytotoxicity (dose range log_10_ –11 to log_10_ –4 M) toward the NCI panel of cell lines from different tumor origins. The 50% inhibition concentrations (log_10_IC_50_) calculated from the dose–response curves are shown in Figure 3A (left side). The values were in a range between –10.49 and –4.93 M.

Apart from the structural descriptions in Chapters 3.1 and 3.2, more general common structural features occurred in relationship to the cytotoxic activity of the 30 anthracyclines quite ubiquitously. There are also structural characteristics typical of representatives with very high—or very low—activities. Such more specific bioactivity-relevant hints come from the ensuing groups, following clear, yet rather gradual, not so strict differences:

Among the top eight compounds, no less than six have tertiary amino groups (**1**, **2**, **3**, **5**, **7**, and **8**), while only few further out of the numerous other, less active compounds have secondary (only compounds **12**, even four-fold, and **14**) or tertiary amino functions (only two compounds, **27** and **30**). All other, mostly less active, amino compounds are primary amines; in the very best active anthracycline, compound **1** (and also in **14**), the amino groups are part of an α-aminonitrile system (a typical Strecker-type aminonitrile), apparently making this functional unit a promising structural feature useful for attaining high cytotoxicity.

Of significant importance for a not-so-good activity seem to be some more special nitrogen-containing functional groups: no less than six of the eleven least active representatives have either an *N*-benzoylhydrazone group (compounds **20** and **21**) or an imino function (compound **22**), an amide group (**23** and **27**) or an oxime entity (**24**), while none of these functional groups are represented in the group of the top-active compounds **1**–**19**.

So, as a preliminary conclusion based on structural considerations, the named, more special *N*-containing groups seem to be disadvantageous for attaining good activity, while the presence of tertiary amines is good for achieving high activities.

In addition to these 30 small-molecule anthracycline derivatives, macromolecular doxorubicin complexes have been tested, viz., doxorubicin bound to DNA (compound **A**), to hydroxyethyl starch (compound **B**), or to transferrin (compound **C**). The log_10_ IC_50_ values are in Figure 3A (right side).

They demonstrate that these doxorubicin complex molecules revealed negligible cytotoxicity toward the cell line panel. The log_10_IC_50_ values ranged between −1.81 and 1.06 M. Therefore, these compounds were not further considered in subsequent analyses. Log_10_IC_50_ values for 30 anthracyclines in a panel of cell lines were also provided (Table S1).

### 3.4. Cross-Resistance to Multidrug-Resistant Tumor Cells with Acquired Resistance

The effectiveness of anticancer drugs is not only determined by their cytotoxic potential as described for 30 anthracyclines above. Anticancer drug activity is frequently hampered by the development of drug resistance. Therefore, we were interested to study the activity of the different anthracycline derivatives in multidrug-resistant tumor cells.

There are two main forms of drug resistance: (1) acquired resistance, which occurs in tumor cells that have been repeatedly exposed to cancer drugs over long periods of time and (2) inherent resistance in tumor cells that have never been in contact with anticancer drugs before. We investigated both types of drug resistance.

We first studied acquired multidrug resistance with a pair of cell lines, viz., OVCAR-8 and NCI-ADR Res ovarian carcinoma cells. Since ovarian cancer is usually treated with anthracyclines, taking OVCAR-8 cells from the NCI tumor panel was a correct choice. This cell line was made resistant by repeated treatments with sublethal concentrations of doxorubicin, finally leading to the establishment of the doxorubicin-resistant subline, NCI-ADR Res.

As can be seen in Figure 3B, *ABCB1* and *ABCB4* were most overexpressed in doxorubicin-resistant NCI-ADR-Res cells compared to parental, drug-sensitive OVCAR-8 cells. The ABCB4 protein is involved in the transport of phosphatidylcholine and other phospholipids and plays a role in hereditary progressive intrahepatic cholestase type 3 (PFIC3). Its expression in doxorubicin-resistant NCI-ADR-Res cells is probably unrelated to the drug-resistance phenotype and could just be the result of non-causative co-expression of this gene during the selection process of drug-resistant cell clones. The expression of all other ABC transporters did not considerably vary between sensitive OVCAR-8 and resistant NCI-ADR-Res cells, indicating that P-glycoprotein/*ABCB1* is the most relevant drug transporter in this cell line and that doxorubicin-resistant NCI-ADR-Res cells are a suitable model for investigating the role of *ABCB1* in cross-resistance for other anthracycline derivatives.

We used the log_10_IC_50_ values of OVCAR-8 and NCI-ADR-Res cells for the anthracyclines to calculate the degrees of resistance. Since the values of NCI-ADR Res cells were only available for 22 of the 30 anthracyclines, the cross-resistance analysis was limited to these 22 compounds. We transformed the log_10_IC_50_ values to linear IC_50_ values and divided those for NCI-ADR-Res cells by those for OVCAR-8 cells. The results are shown in Figure 3C. The degrees of resistance for 20 of the 22 anthracyclines varied from 160.34 to 1.05. High degrees of resistance were obtained to clinically established drugs: 160.34-fold for doxorubicin (**13**), 121.0-fold for daunorubicin (**9**), 104.19-fold for mitoxantrone (**12**), 40.56-fold for epirubicin (**19**), and 18.18-fold for idarubicin (**4**). The NCI-ADR-Res cells were less or even negligibly cross-resistant to the other novel derivatives.

Interestingly, the IC_50_ values of OVCAR-8 cells for compounds **26** and **1** were even higher than those for NCI-ADR-Res cells, indicating that the otherwise NCI-ADR-Res cells were hypersensitive to these two compounds. This hypersensitivity phenomenon is termed collateral sensitivity. The degrees of collateral sensitivity (= inverse degrees of resistance) of OVCAR-8 were 3.61-fold for compound **26** and 50.35-fold for compound **1** (Figure 3C, right side).

### 3.5. Structure–Activity Relationships Regarding the Cross-Resistance to Multidrug-Resistant Tumor Cells

As a next step, we considered the relationship between the resistance degrees and the structural features of the 22 anthracyclines. No less than 14 of the compounds with the highest degree of resistance are all primary amines—there are among them only two compounds with secondary amino functions (**12**, which has even four such groups, and **14**), but none with a tertiary amino group, while the situation is opposite for the less resistant or even non-resistant representatives:

All six representatives that have a tertiary amino function (compounds **1**, **2**, **3**, **7**, **27**, and **30**) were among the 13 agents with the lowest resistance values, showing that tertiary amino groups are favorable for avoiding resistance formation, while primary ones rather increase resistance tendencies.

Of particular value is 3′-deamino-3′-(4″-(3″-cyano)morpholinyl)-doxorubicin (**1**), which even shows a strong collateral activity—while being (as the term “compound **1**” indicates) the one with the lowest log_10_IC_50_ value out of all 30 compounds. For comparison, the log_10_IC_50_ value of **1** was three orders of magnitude lower (–10.49 M) than that of doxorubicin (**13**) as the parent compound (−7.24 M). The strong cytotoxicity and the fact that multidrug-resistant cells were collaterally sensitive to this drug indicates that compound **1** may be especially suited to treat ABCB1-expressing tumors with much better efficacy than the parent compound, doxorubicin. One characteristic structural feature of compound **1** is that it is not just a normal tertiary amine, but part of an α-aminonitrile (a typical Strecker-type compound). The only other compound with an α-aminonitrile unit is compound **14**, exhibiting a low, not-too-bad resistance factor of 26.79, showing that an aminonitrile function, in particular in combination with a tertiary amino part (as in **1**), is a highly promising structural entity.

Very special were the following three compounds, which were outliers from a structural point of view: Compound **12** has no amino sugar entity at all (and no tertiary or secondary amino function), but open-chain substituents with no less than four secondary amino groups. It is the only agent with a completely symmetric structure; it is an interesting structural alternative, but in this series a true outlier. Compound **26** has no amino sugar, and (together with compound **29**, see below) it is the only compound that has no nitrogen at all, but instead a nitrogen-free deoxy sugar, and shows a very low degree of resistance. Compound **29** is likewise very special. It has no nitrogen, and, as an aglycone, even possesses no sugar. It will be of great interest to investigate why these latter compounds were so active, although their structures diverge from the narrow standard given by the majority of the other investigated compounds.

### 3.6. Resazurin Assay Results

**Figure 4:**
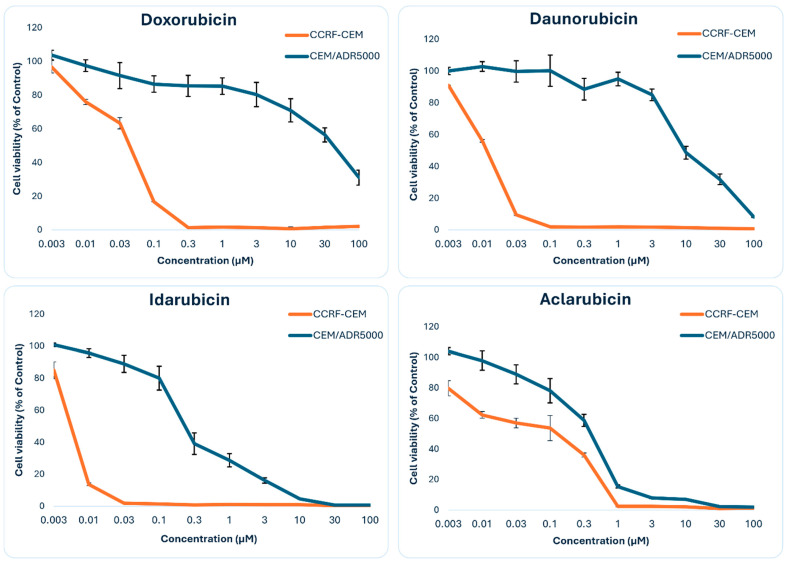
Resazurin cell viability assay. Dose–response curves of doxorubicin, daunorubicin, idarubicin, and aclarubicin with the sensitive CCRF-CEM and multidrug-resistant P-glycoprotein overexpressing CEM-ADR5000 leukemia cells for 72 h. The data represents the mean ± standard deviation from three independent experiments.

The analysis of the cytotoxicity assay gave the IC_50_ values shown in Table 2. The degrees of resistance (IC_50_ of CEM/ADR5000/IC_50_ of CCRF-CEM) for doxorubicin (compound **13**) and daunorubicin (compound **9**) were high (988- and 824-fold, respectively). CEM/ADR5000 cells were intermediate resistant toward idarubicin (compound **4**, 46-fold), while aclarubicin (compound **7**) yielded only low resistance (2.6-fold) in CEM/ADR5000 cells (Figure 4).

### 3.7. Live Cell Time-Lapse Imaging

**Figure 5:**
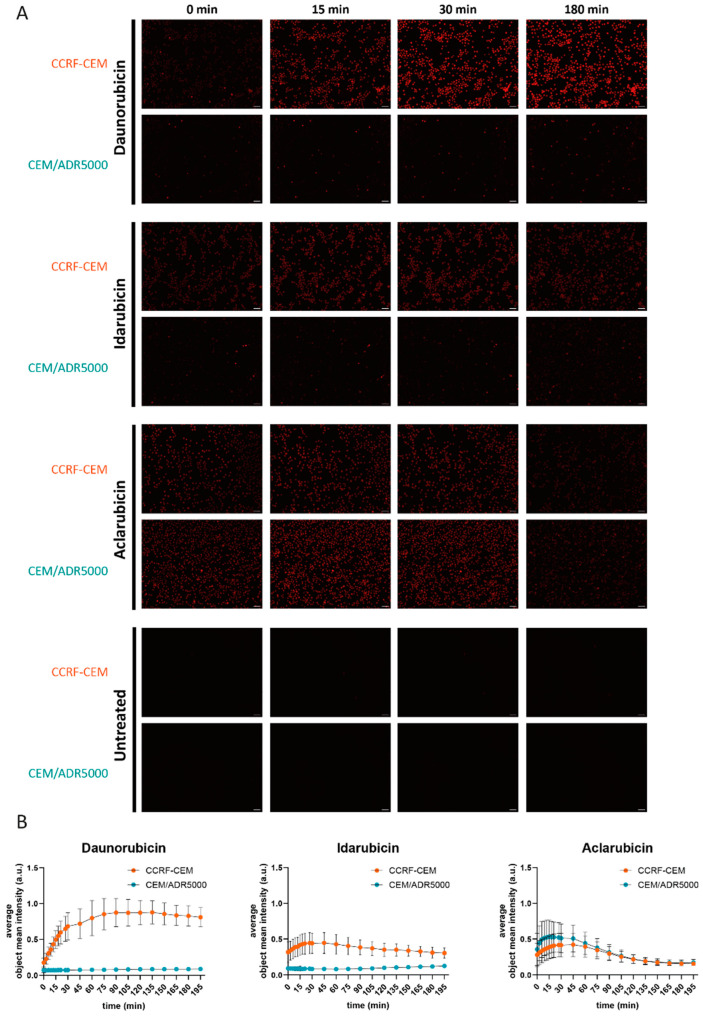
Live cell time-lapse imaging of CCRF-CEM and CEM/ADR5000 cells upon treatment with selected anthracyclines. (**A**) and Figure S1 show representative images of CCRF-CEM and CEM/ADR5000 cells treated with the indicated compounds (from top to bottom, daunorubicin (**9**), idarubicin (**4**), aclarubicin (**7**), and untreated cells). The results of CCRF-CEM cells are shown in the first row, and those for CEM/ADR5000 cells are shown in the second row. Fluorescent images and merged images of fluorescence signal and phase contrast (Figure S1) are shown at 0 min, 15 min, 30 min, and 3 h after compound addition. The scale bar in merged images corresponds to 50 µm. (**B**) Quantification of cellular uptake of daunorubicin, idarubicin, and aclarubicin in CCRF-CEM cells (orange) and CEM/ADR5000 cells (green) was carried out by the built-in analysis module, and the average object mean intensity was plotted. Error bars show standard deviation of three biological replicates. Time lapses were acquired every 3 min for the first 0.5 h and every 15 min for the following 2.5 h. At each time point, daunorubicin and idarubicin were significantly accumulated at higher levels in CCRF-CEM cells compared to CEM/ADR5000 cells (unpaired *t* test, *p* < 0.0001), whereas aclarubicin accumulated at a similar level in both cell lines (the unpaired *t* test showed no significant difference, *p* > 0.0654).

We employed the intrinsic fluorescence of anthracycline compounds [45] to determine whether the compounds are substrates of P-glycoprotein. Specifically, we compared the kinetics of the cellular uptake among compound **7** (aclarubicin), compound **4** (idarubicin), and compound **9** (daunorubicin), selected for their low, intermediate, and high cross-resistance profiles, respectively (Figure 3C), by determining the differences in accumulation of fluorescence intensity immediately upon adding the compound up to 3 h later. Figure 5A,B show the differences in the cellular uptake between CCRF-CEM and CEM/ADR5000 cell lines among the three compounds, which reflects their cross-resistance profiles. As anticipated, daunorubicin uptake by the CCRF-CEM cells was significantly higher than in the case of the P-glycoprotein expressing CEM/ADR5000 cells (*p* < 0.0001). Idarubicin accumulated with slower kinetics compared to daunorubicin (as shown by the lower fluorescence mean intensity values), although still showing statistical difference between the two cell lines. Aclarubicin uptake did not differ significantly, indicating no impact of P-glycoprotein on its efflux inside the cells, confirming its low cross-resistance (Figure 3C).

### 3.8. Correlation of IC_50_ Values and P-Glycoprotein/MDR1 Expression

As shown above, the investigation of anthracycline cross-resistance in doxorubicin-resistant NCI-ADR-Res cells in comparison to parental, drug-sensitive OVCAR-8 cells pointed to the role of *ABCB1* for acquired resistance. Next, we also studied the role of *ABCB1* in cell lines that were not selected for drug resistance to explore the role of *ABCB1* for inherent drug resistance.

For this reason, we correlated the mRNA expression of *ABCB1* in 58 NCI tumor cell lines not preselected for drug resistance with the log_10_IC_50_ expression for our set of 30 anthracycline derivatives. The mRNA expression was determined using qRT-PCR and microarray hybridization with two arrays (U95Av2A-E and U133A/B). In addition, we also analyzed the expression of three onco-miRs (miR-451, has-miR-27aN, and miR-027a-prec as determined by qRT-PCR), which are known to regulate the ABCB1/P-glycoprotein expression. Furthermore, we also included data on the relative amplification of the chromosomal locus 7q21, where the *ABCB1* gene is located, to study the role of *ABCB1* gene amplification for the cellular response to the 30 anthracyclines.

We first correlated the expression of *ABCB1* and three onco-miRs in the 58 cell lines to the log_10_IC_50_ values. These analyses were based on a matrix of 12,180 single datapoints (=58 cell lines × 30 anthracyclines × 7 mRNA/onco-miR/DNA parameters). Applying the Pearson correlation test delivered us significance values (*p*) and correlation coefficients (*r*).

**Figure 6:**
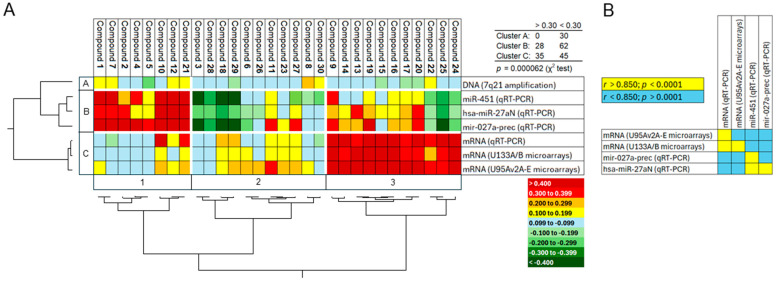
Correlation of cellular response to 30 anthracyclines and expression of *ABCB1* mRNA and P-glycoprotein/*ABCB1*-regulating onco-miRs in 58 tumor cell lines with inherent resistance. The *ABCB1* expression has been measured using qRT-PCR and microarray hybridization with U95av2A-E and U133A/B arrays. The expression of onco-miRs miR-451, miR-027a prec, and miR-27aN has been detected using qRT-PCR. (**A**) Cluster image map displaying the correlation between cellular response to anthracyclines and *ABCB1* mRNA and onco-miR expression of 49 ABC transporter genes in 58 tumor cell lines with inherent resistance. The correlations have been determined by Pearson’s correlation test. The correlation coefficients (*r*-values) have been subjected to hierarchical cluster analysis (Ward method) and color-coded. The dendrograms obtained for the clustering of the anthracyclines and the *ABCB1* mRNA and onco-miR expression have been used to construct a cluster image map. The separation in cluster regions was statistically significant (*p* = 0.000062, χ^2^ test). (**B**) Color-coded correlation map between three parameters to measure *ABCB1* mRNA expression (qRT-pCR and two microarrays) and three onco-miRs by qRT-PCR. Yellow boxes indicate correlations with correlation coefficients of *r* > 0.850 and *p* < 0.00001 and blue boxes of *r* < 0.850 and *p* > 0.0001.

The *r*-values of 210 (=30 × 7) correlations were then subjected to hierarchical cluster analysis. We clustered along the 30 anthracyclines and the seven *ABCB1*/onco-miR parameters. The dendrograms obtained from both cluster analyses were then used to generate a cluster image map, where the *r*-values were color-coded (Figure 6). This cluster image map clearly illustrates that the overlap quantities of cluster B and cluster 2 as well as cluster C and cluster 3 were most interesting. These two cluster hotspots mostly consisted of *r*-values higher than 0.3. In cluster region B/1, compounds **1**, **7**, **2**, **4**, **5**, **10**, **12**, and **21** were correlated with the three mRNA expression parameters. In cluster region C/3, compounds **9**, **14**, **13**, **19**, **15**, **16**, **17**, **20**, **22**, **25**, and **24** were associated with the expression of the three onco-miRs. All other cluster regions (A/1, A/2, A/3, B/2, B/3, C/1, and C/2) showed lower *r*-values or displayed even inverse correlations (*r* < −0.2). Cell lines with strong correlations to *ABCB1* mRNA expression showed weak associations to onco-miR expression and vice versa. The degree of gene amplification of chromosomal locus 7q21 was consistently low in all cell lines, indicating that *ABCB1* gene amplification was of minor importance in this set of cell lines. We subjected all results to the χ^2^ test and found a statistically significant distribution across the cluster image map (*p* = 0.000062) (Figure 6A, upper right corner).

Additionally, we correlated the data obtained from qRT-PCR and the two different microarrays and found strong relationships (*r* > 0.85; *p* < 0.0001). Similarly, we correlated the expression of the three onco-miRs and also found strong correlations (Figure 6B). These results speak for the validity of the ABCB*1 and* onco-miR expression profiles in the 58 tumor cell lines.

### 3.9. Correlation of IC_50_ Values and All ABC Transporters

So far, our investigations focused on P-glycoprotein/*ABCB1*. However, *ABCB1* is only one out of 49 members of the ABC transporter family. Hence, we wanted to find out whether other members of this gene family might also contribute to resistance to the 30 anthracycline derivatives.

**Figure 7:**
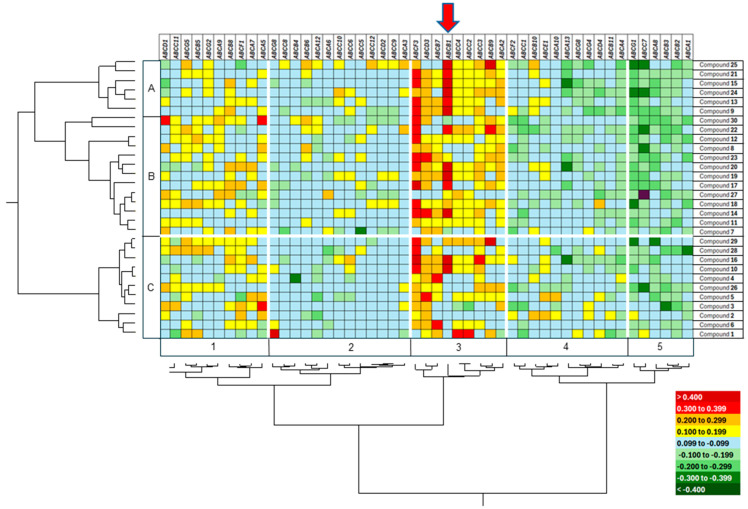
Cluster image map displaying the correlation between cellular response to 30 anthracyclines and mRNA expression of 49 ABC transporter genes in 58 tumor cell lines with inherent resistance. The correlations have been determined by Pearson’s correlation test. The correlation coefficients (*r*-values) have been subjected to hierarchical cluster analysis (Ward method) and color-coded. The dendrograms obtained for the clustering of the anthracyclines and the ABC transporter genes have been used to construct a cluster image map. The anthracyclines clustered in two main cluster branches (A, B) and the ABC transporter genes in five main branches. Cluster 3 contained ABC transporter genes that correlated with higher *r*-values of cellular resistance to anthracyclines than others. Of them, *ABCB1* was the most prominent one (see arrow). The tumor cell lines were not pre-exposed to anticancer drugs and, therefore, represent a suitable model for inherent drug resistance.

Therefore, we correlated the mRNA expression of the NCI cell line panel for 49 ABC transporters with the log_10_IC_50_ values of the 30 anthracycline derivatives using Pearson correlation tests. For this analysis, we did not use NCI-ADR cells, which were selected for doxorubicin resistance, and MDA-N cells, which were transfected with a cDNA coding for *EGFR* (epidermal growth factor receptor) because we wanted to study the role of ABC transporters only in inherently resistant cell lines. The results were subjected to hierarchical cluster analysis. As can be seen in Figure 7, the expression of *ABCB1*, *ABCD3*, and *ABCF3* was most frequently correlated with the log_10_IC_50_ values of anthracyclines. ABCD3 is a fatty acid-CoA transporter and the ABCF3 protein is a lipid transporter, both of which do not transport cancer drugs. Hence, their correlations to anthracyclines may not be causative and not related to drug resistance, indicating that P-glycoprotein/ABCB*1 was* the most important ABC transporter mediating inherent resistance to anthracyclines. Interestingly, other ABC transporter genes that have been described to confer resistance in cell lines with acquired doxorubicin (e.g., *ABCC1*, *ABCC2*, and *ABCG2*) were not related to inherent anthracycline resistance in this panel of tumor cell lines.

### 3.10. Molecular Docking of 30 Anthracyclines to P-Glycoprotein

The structure–activity relationships may become more rational and understandable if they are accompanied by molecular docking experiments. The significant correlations between the cellular response to anthracyclines and *ABCB1* gene expression indicate a causative role for *ABCB1*. To prove this hypothesis in more detail, we performed molecular docking experiments using the three-dimensional crystal structure of P-glycoprotein, which is encoded by the *ABCB1* gene, to study the binding of the 30 anthracycline derivatives of this drug pump.

As shown in Table 3, the lowest binding energies (LBE) ranged from −17.5 (±0.5) to −9.5 (±<0.0 1) kcal/mol. The parental compounds doxorubicin (**13**) and daunorubicin (**9**) had intermediate LBE values, viz., −12.2 (±0.01 ) and −10.9 (±0.1), respectively. Elacridar, as a known P-glycoprotein-binding and -inhibiting compound, was used as a positive control and displayed a binding energy of −14.3 (±0.1) kcal/mol. The predicted inhibition constants (pK_i_) showed low nanomolar concentrations between 0.18 (±0.13 ) × 10^−3^ nM for compound **27** and 29.1 (±9.2) nM for compound **12**. Elacridar had a pK_i_ value of 35.49 (±3.5) × 10^−3^ nM (Table 2). Most compounds interacted with nine to twelve amino acid residues.

**Figure 8:**
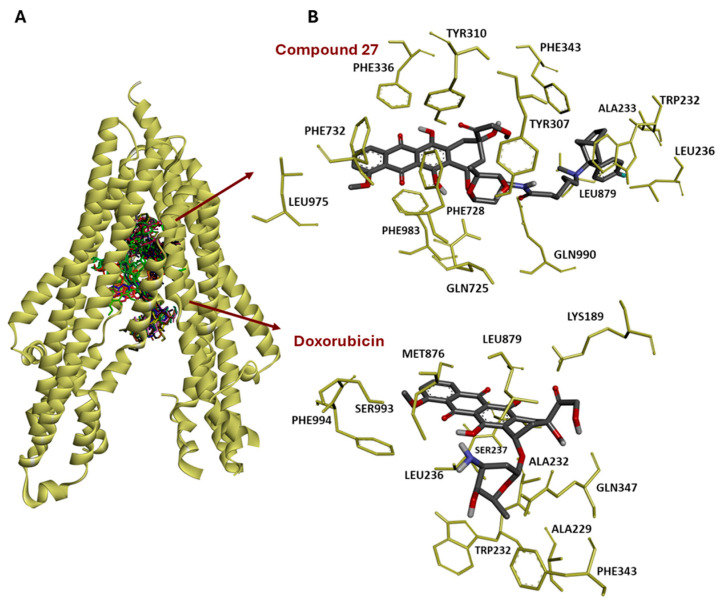
Molecular docking of 30 anthracyclines and elacridar (positive control) to P-glycoprotein/ABCB1 (PDB: 8Y6H). (**A**) The compounds bound to a domain at the inner side of P-glycoprotein which serves as a channel by which compounds are extruded out of the cell. (**B**) The zoomed-in view on the right side shows the interaction between compound **27** and doxorubicin as the parent compound.

We visualized the binding of the 30 anthracyclines and elacridar. All compounds interacted with the same binding domain at the inner side of the efflux channel of P-glycoprotein (Figure 8A). The binding of doxorubicin as the parent compound and compound **27** is exemplarily shown at the right side of Figure 8B.

### 3.11. Structure–Activity Relationships Regarding Molecular Docking of the 30 Anthracyclines to P-Glycoprotein/ABCB1

**Figure 9:**
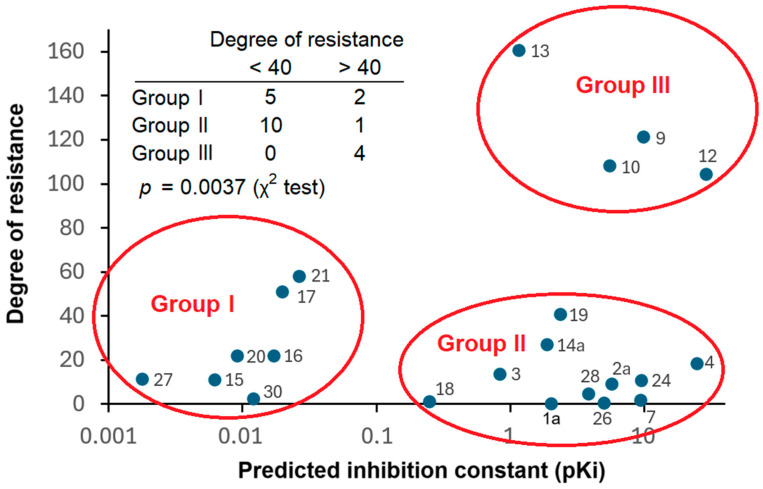
Correlation of degrees of resistance and predicted inhibition constants (pK_i_) of 22 anthracyclines. The degrees of resistance were calculated from the IC_50_ values of multidrug-resistant NCI-ADR Res cells compared to parental, drug-sensitive OVCAR-8 (see Figure 3B). The pK_i_ values were determined by molecular docking of the anthracyclines to P-glycoprotein (see Table 3). The separation of the anthracyclines into the three different Groups I to III was statistically significant. (*p* = 0.0037; χ^2^ test).

We correlated the degrees of resistance in NCI-ADR-Res cells of the 22 anthracyclines with the predicted inhibition constants (pK_i_) obtained from molecular docking to P-glycoprotein. As can be seen in Figure 9, the compound can be separated into three different groups with statistical significance (*p* = 0.0037; χ^2^ test): Group I with low degrees of resistance and low pK_i_ values, Group II with low degrees of resistance but high pK_i_ values, and Group III with high degrees of resistance and high pK_i_ values. Group I consisted of seven compounds, Group II was composed of eleven compounds, and Group III had four compounds, making a total of twenty-two compounds.

The structural differences between the three distinct groups showed a clear tendency regarding the number and character of nitrogen functions and OH groups:

**Group I** (seven compounds): Four out of seven compounds (**27**, **20**, **16**, **21**) have more than one nitrogen atom (two compounds, **20** and **21**, even have three), significantly more than in any other group; the additional N atoms occur in the form of amides (**27**), hydrazones (**20**, **21**), and a pyridine ring (**16**); all these entities were found only in this group. All compounds in this cluster have two aliphatic OH groups; only **27** has three.

**Group II** (11 compounds): In this group, nearly all compounds possess just one (basic) nitrogen atom, only 3/11 compounds (**1a**, **14a**, and **24**) have two nitrogen atoms (in the form of aminonitriles and an oxime, all only in this group), one compound (**26**) has no nitrogen at all. Here, a majority of representatives (6/11, compounds **3**, **1a**, **14a**, **19**, **26**, **2a**) have three (in the case of **2a**) or even four aliphatic OH groups; only five compounds have only two aliphatic OH groups.

**Group III** (four compounds): Only in this group, out of a total of all twenty-two grouped compounds, there is one (**12**) that has four secondary amino functions, but it has to be considered as a true outlier; as the only one out of twenty-two compounds, compound **12** has no sugar residue.

Concerning the interactions of the different amino acids with different anthracyclines, we made some significant observations distinguishing the compounds of the Groups I to III from each other:

(1) The 52 amino acids involved interacted in most different ways: some (less frequently occurring ones) were involved only for just one of the compounds (in the cases of Gln 195, Ile 235, Thr 240, Ala 295, Ile 306, Ser 344, Phe 372), but there was also one, Phe 983, which was interacting with nearly *all* compounds (21/22 compounds in the three clusters). Thus, it played a central role.
(2) Likewise, most divergent were the numbers of amino acids interacting with representatives of the three different groups, extremes being some amino acids specifically did not interact with members of *all* three groups, but only with compounds from one or two groups, with examples being Gln 195, Ile 235, Ser 237, Phe 239, Thr 240, Ala 295, Ile 306, Leu 339, and Ile 340, Ser 344, Lys 877, Ser 880, Ser 993, which interact with none of the compounds from Group I.

On the contrary, other amino acids, such as Met 69, Gln 195, Ile 299, Ser 344, Pro 350, Phe 728, Leu 975, Phe 978, Ser 979, Gln 990, and several more (in total 27) interacted with none of the Group III compounds. In a similar way, some amino acids (among them Ile 299) did not interact with any of the Group II compounds. As a consequence, some amino acids (namely, Gln 195, Ser 344, Phe 770, and others (in total six)) interacted only with compounds from Group I or Group II (in total seven) or Group III (in total only one, Phe 770), thus contributing to further demarcating the three Groups I, II, and III from each other and, hence, they justified the Group I to III classification.

(3) Particularly noteworthy is the behavior of the three pairs of diastereomers of compounds **1a/b**, **2a/b**, and **14a/b**, although they differed only by the absolute configuration at only one out of seven chiral centers, and all bound with energies that differed by only 0.3 kcal/mol between the respective diastereomers:
  • **1a** bound to ten amino acids, of which three (Tyr 307, Ile 340, Phe 732) did not bind to **1b**, while **1b** bound to eleven amino acids, of which four (Tyr 310, Phe 336, Leu 339, Phe 728) did not bind to **1a**. The other interactions were identical.
  • In a similar way, **2a** bound to ten amino acids, of which two (Tyr 932, Phe 978) did not bind to **2b**, while **2b** bound to nine amino acids, of which one (Tyr 953) did not bind to **2a**. The other interactions were identical.
  • In a sharp contrast, **14a**/**14b** differed dramatically from each other: **14a** bound to nine amino acids, of which *none* was identical to any of the eight amino acids to which its diastereomer, **14b**, bound.

This emphasizes, once again, the importance of considering each single stereogenic element (here in particular the chiral center at C-1″ of **14a/b**), even though the resulting binding energies look so similar.

In general, the anthracyclines were found to interact with usually about ten amino acid residues—from eight amino acids (from compounds **6**, **14b**, and **28**) up to fourteen amino acids (for compounds **15**, **20**, and **27**).

An extreme exception is mitoxantrone (compound **12**), with only four amino acid contacts. It is a true outlier, including structurally, being a tri-, not tetracyclic, and fully symmetric compound, without any stereogenic center and without a sugar moiety, but having, as the only one out of 30 compounds, four secondary amino groups.

### 3.12. Kaplan–Meier Survival Analysis

Given the relevance of ABC transporters for resistance to anthracycline derivatives, the question arises about the prognostic role of ABC transporters for the survival of cancer patients. In the past, this issue has been repeatedly addressed with controversial results. Therefore, we re-addressed this important question.

The correlation analyses between the log_10_IC_50_ values of the NCI cell line panel for 30 anthracycline derivates revealed *ABCB1* as the most important resistance factor. For this reason, we only focused on *ABCB1* in the survival analysis. By analyzing the tumor repository of The Cancer Genome Atlas (TCGA) (www.cancer.gov/ccg/research/genome-sequencing/tcga and www.kmplot.com/analysis/, accessed on 1 November 2024), we performed Kaplan–Meier survival analyses for 23 different tumor types with 7489 tumor biopsies.

**Figure 10:**
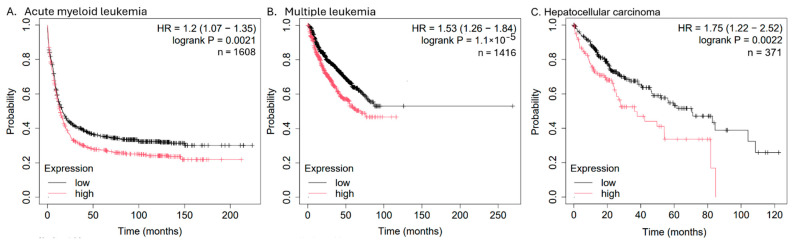
Kaplan–Meier statistics of overall survival times of patients suffering from (**A**) acute myeloid leukemia, (**B**) multiple myeloma, or (**C**) hepatocellular carcinoma.

The *ABCB1* mRNA expression significantly correlated with shorter overall survival in acute myeloid leukemia (*n* = 1608), multiple myeloma (*n* = 1416), and hepatocellular carcinoma (*n* = 371) (Figure 10) but not in other cancer types (carcinomas of the bladder, breast, esophagus, head and neck, kidney, lung, ovary, pancreas, rectum, stomach, thyroid, or uterus; pheochromocytoma and paraganglioma, testicular germ cell tumors, and thymoma). Since anthracyclines are clinically used to treat multiple myeloma, acute myeloid leukemia, and hepatocellular carcinoma, *ABCB1* may serve as a prognostic factor for the survival probability of patients affected with these tumor types.

## 4. Discussion

Anthracyclines were developed back in the 1960s and 1970s and have been an integral part of chemotherapy for many types of hematological and solid tumors ever since [46]. To this day, they are indispensable in clinical oncology despite certain disadvantages, e.g., the development of resistance and severe side effects (especially cardiotoxicity). In the present study, we focused on the resistance problem. The multidrug resistance (MDR) phenomenon is a major problem that affects anthracyclines in particular, along with other classes of anticancer drugs. MDR is mediated by various ABC transporters, of which P-glycoprotein (*ABCB1*, *MDR1*), MDR-related proteins 1 and 2 (*ABCC1/2*, *MRP1/2*), and breast cancer resistance protein (*ABCG2*, *BCRP*) are the best-studied representatives of this gene family [47].

### 4.1. Structure–Activity Relationships

The clinically established anthracyclines (doxorubicin, daunorubicin, epirubicin, idarubicin) are all transported by P-glycoprotein. This is why it is so important to find new anthracyclines that do not cause cross-resistance in doxorubicin-resistant, P-glycoprotein-expressing tumor cells. The studies have shown that favorable structural properties for achieving high cytotoxicity for anthracyclines are the presence of tertiary amino groups, in particular if being part of an α-aminonitrile unit, while compounds with primary amino groups and also special nitrogen-containing units such as hydrazones, oximes, imines, and amides were found among the less active representatives.

Great attention must be also attributed to avoiding the development of drug resistance. Here again, the presence of tertiary amino functions was helpful in avoiding resistance, while primary amines rather increased the danger of resistance development. Again, the combination with a nitrile in the form of an α-aminonitrile function as in 3′-deamino-3′-(4″-(3″-cyano)morpholinyl)-doxorubicin (**1**) was favorable, helping to make this particular compound both the most active and least resistance-supporting compound of all agents tested in this study. 3′-Deamino-3′-(4″-(3″-cyano)morpholinyl)-doxorubicin (**1**) was also remarkable because multidrug-resistant NCI-ADR Res cells were even more sensitive to this compound than the parental drug-sensitive OVCAR-8 cell line.

The mechanism of collateral sensitivity is well known for some anticancer drugs, as first described by Bech-Hansen (1976), for synthetic as well as natural compounds [48,49,50]. Mechanisms involved in collateral sensitivity include the overwhelming of the ATP demands of P-glycoprotein, inhibition of glutathione-related enzymes, and alternative signaling pathways [51,52,53]. While resistance of other cytotoxic compounds to doxorubicin has been previously observed [54], collateral sensitivity of a doxorubicin-resistant cell line to another anthracycline derivative is uncommon and has not been reported before to the best of our knowledge.

### 4.2. ABC Transporters in Acquired and Inherent Resistance

Doxorubicin-resistant NCI-ADR-Res cells have been generated by exposure to doxorubicin and display a multidrug-resistance profile with overexpression of P-glycoprotein and cross-resistance to cisplatin, paclitaxel, docetaxel, gemcitabine, etoposide, and the ferroptosis inhibitor erastin [32,55]. In the present investigation, we observed cross-resistance to several but not to all of the 30 anthracyclines, as discussed above. Inspecting the expression of all other ABC transporters of this gene family showed low expression of all other ABC transporters except for *ABCB4*. The ABCB4 protein is, however, not involved in the transport of anticancer drugs or other xenobiotic compounds. Instead, it transports phosphatidylcholine and other phospholipids and plays a role in hereditary progressive intrahepatic cholestase type 3 (PFIC3). This indicates that P-glycoprotein/*ABCB1* was the most important MDR-conferring ABC transporter for acquired cross-resistance to anthracyclines.

To further verify the role of P-glycoprotein for the 30 anthracyclines, we performed molecular docking. All anthracyclines bound with high affinity to the same domain at the inner channel site of P-glycoprotein as elacridar, and elacridar did not bind with considerably lower LBE and pK_i_ values. Elacridar is well-known to bind and inhibit P-glycoprotein [56] and was, therefore, used by us as a positive control. Our results indicate that P-glycoprotein is indeed an important interaction partner not only for established anthracyclines such as doxorubicin and daunorubicin but also for the other new anthracyclines. It was unexpected, however, that compound **1**, which showed the highest cytotoxicity in the NCI cell line panel and exhibited collateral-sensitive features in multidrug-resistant cells, did not display the lowest LBE and pK_i_ values among the anthracyclines tested. It can be speculated that the activity of compound **1** may not be solely determined by P-glycoprotein but that other mechanisms of cytotoxicity may be also operative, such as inhibition of DNA topoisomerase II and DNA double-strand breaks, DNA intercalation, and generation of reactive oxygen species as described for doxorubicin and its derivatives [57]. Collateral sensitivity can be caused by P-glycoprotein-dependent and -independent mechanisms. Here, modes of action other than P-glycoprotein may be hypothesized.

In the past, cancer cell lines were frequently used in which multidrug resistance was induced by drug exposure. Here, the importance of P-glycoprotein and other ABC transporters has been well established [58], and the current analyses also indicate the importance of these efflux pumps. The importance of ABC transporters has been less well studied in cell lines with inherent resistance, probably because the relationships are less clear and therefore more difficult to detect. This is possibly because no selection of certain dominant factors took place in the case of inherent resistance and therefore many other mechanisms, which are already present in cancer cells, compete for the expression of drug resistance. Various factors have been discussed, such as mutations in oncogenic and tumor suppressor genes [59,60], which not only play a role in carcinogenesis but also in the response to anticancer drugs. Furthermore, the detoxification of xenobiotic substances, which include anticancer drugs, by hepatic phase I/II/III metabolism plays an important role [61,62]. The efficacy of anticancer drugs that damage the DNA of tumor cells can be influenced by DNA repair mechanisms [63,64]. In addition, mechanisms of programmed cell death have also been intensively studied in recent years in connection with drug resistance. In addition to non-surgical mechanisms of apoptosis, autophagy, ferroptosis, mitophagy, necroptosis, and other cell death pathways play a role in preventing tumor cells from initiating cell death despite severe lesions caused by anticancer drugs [65]. More recently, cellular senescence and quiescence have also been described as mechanisms of resistance development [66,67].

Our analysis of 49 ABC transporters revealed ABCB1 as the most important ABC transporter in the panel of cell lines not preselected for drug resistance. Other ABC transporters of at least some relevance for resistance to the 30 anthracyclines were ABCC2-5, which are also known to mediate MDR. Surprisingly, other drug pumps with known functions in MDR were of minor importance, e.g., ABCC1 and ABCG2. Instead, ABC transporters not related to anticancer drug resistance were associated with cellular response to the 30 anthracyclines, viz., *ABCF3*, *ABCD3*, *ABCB7*, *ABCB9*, and *ABCA2*. The ABCF3 protein transports nucleotides and nucleosides and exerts anti-flaviviral effects. The ABCD3 protein transports bile acids, fatty acids, and fatty acyl-CoAs from the cytosol into the peroxisomal lumen. This transporter is important for peroxisome biogenesis and β-oxidation. ABCB7 transports heme and iron–sulfur cluster precursors from mitochondria to the cytosol. The ABCB9 protein translocates a broad spectrum of peptides from the cytosol into the mitochondria [10,12]. Given these diverse functions, we do not assume that these transporters are of much relevance for anthracycline treatment, as there is no direct evidence that they transport anticancer drugs at all.

Our findings emphasize the dominant role of P-glycoprotein/MDR1 *in* the response rate of tumors to anthracyclines. The literature reports that other ABC transporters (ABCC subfamily members, ABCG2) that may also be important in the development of doxorubicin resistance [47] were not confirmed in our analysis of the NCI tumor panel. However, we were able to show that P-glycoprotein/ABCB1 is important for both acquired and inherited forms of resistance.

In addition to the chemical investigation of anthracyclines, concepts based on nanotechnology were launched for the generation of doxorubicin nanocomplexes. For instance, transferrin has been used as a carrier for doxorubicin [68]. Since cancer cells express more transferrin receptors than normal cells [69,70] and the activity of doxorubicin can be increased by ferrous iron in a Fenton-type reaction [71], doxorubicin–transferrin complexes have been considered to overcome drug resistance and induce ferroptosis as an iron-dependent mode of programmed cell death [72]. Furthermore, hydroxyethyl starch has been used as a nanocarrier for doxorubicin [73]. Another approach was the preparation of doxorubicin coupled with DNA fragments [74]. Therefore, we also included doxorubicin complexed with transferrin, hydroxyethyl starch, or DNA fragments in our analyses. Unfortunately, all three nanocomplexes showed weak activity toward the NCI cell line panel, and we did not continue investigating them further.

The advantages of investigating different mechanisms in specific suitable cell models may explain why there are considerably more in vitro results than clinical data. The complexity of the resistance mechanisms makes it difficult to analyze them in clinical tumors. It was therefore important for us to analyze the prognostic relevance of ABC transporters in clinical tumor biopsies and to investigate their significance for the survival probability of tumor patients. This has already been reported by many groups in the past. However, contradictory results have often been published in the synopsis, so that the actual prognostic significance of ABC transporters is still unclear today.

A tremendous progress in identifying prognostic biomarkers was the sequencing of cancer genomes in The Cancer Genome Atlas (TCGA) project. Large amounts of data were collected without initially focusing on specific genes but instead identifying genome-wide expression and mutation profiles for many different tumor types. This provides a non-biased database that was generated according to standardized criteria. The Kaplan–Meier analysis of the survival time of patients with 23 different tumor types showed that *ABCB1* expression is prognostically relevant for leukemia and multiple myeloma. Here, we found that the high expression of *ABCB1* was significantly correlated with short overall survival times of patients.

### 4.3. Prognostic Relevance of P-Glycoprotein/ABCB1 and Diagnostic/Therapeutic Implications

There were numerous reports from the 1980s and 1990s investigating the prognostic value of P-glycoprotein/*ABCB1* in many tumor types. It has been described that P-glycoprotein/*ABCB1* is linked to worse survival prognosis [23,24,75], including leukemia, multiple myeloma, and hepatocellular carcinoma albeit with low patient numbers [76,77,78,79]. However, there are also contradictory reports, and it is not established as a clinical routine biomarker for drug resistance in leukemia and other tumor types [80,81]. Therefore, we re-analyzed this important question by taking advantage of TCGA database. The fact that we found significant results for leukemia, multiple myeloma, and hepatocellular carcinoma but not for other tumor types may have important implications for future research:

• P-glycoprotein/*ABCB1* could be used as a prognostic marker in these two tumor types
• P-glycoprotein/*ABCB1* expression may serve as a biomarker for individualized therapy with anthracyclines (and other MDR-related drugs)
• P-glycoprotein/*ABCB1* may serve as a target protein for inhibitors of the efflux function and the development of collaterally sensitive drugs.

In addition to pharmacological interventions by non-cross-resistant or collateral-sensitive anthracyclines as outlined in the current study, it was previously also attempted to develop diagnostic tests to detect pre-therapeutic MDR for individual chemotherapeutic regimens. The purpose was to individually adapt the chemotherapeutic regimen with anthracyclines (and also other MDR-related drugs) according to the expression of ABC transporters. However, such predictive tests failed clinical establishment for routine diagnostics because of methodological deficiencies [82,83]. The advent of novel techniques such as RNA sequencing, which is much more precise than older methods (immunohistochemistry, non-quantitative PCR), merit a re-evaluation of this concept. Our results clearly indicate that RNA sequencing-based measurement of *ABCB1* mRNA expression levels is important for anthracycline resistance. Hence the development of diagnostic tests of *ABCB1* expression may facilitate attempts for individualized cancer treatments.

Huge efforts have been undertaken to identify pharmacological inhibitors for these ABC transporters in order to overcome or reverse MDR and to re-sensitize human tumors to chemotherapy [84,85,86,87]. Several clinical trials have been performed to test MDR-inhibiting substances for their capability to sensitize refractory human tumors in the clinical setting. For diverse reasons, these clinicals trials failed, as discussed elsewhere, and no MDR inhibitor reached clinical approval [88,89]. A recent clinical trial, however, reported an interesting revival. The combination of paclitaxel with the P-glycoprotein inhibitor encequidar led to a significantly better treatment response than paclitaxel alone and a tendency for prolonged survival times in a clinical Phase-III trial with metastatic breast cancer [90]. Nevertheless, it seems that the development of P-glycoprotein/*ABCB1* inhibitors is not a trivial task. In this context, it may be more straightforward to develop novel drugs which are not transported by P-glycoprotein/ABCB*1 and* therefore bear the potential to kill multidrug-resistant tumor cells with similar efficacy to otherwise drug-sensitive cells. The non-cross-resistant anthracyclines described in the present paper may be a starting point for novel drug developments.

## 5. Conclusions

Among 30 anthracyclines, compound **1** was most favorable because it had the highest in vitro cytotoxic activity, and multidrug-resistant cells displayed collateral sensitivity to this substance. Among 49 ABC transporters, P-glycoprotein/*ABCB1* was the most important one for acquired or inherent resistance to these anthracyclines. Molecular docking demonstrated that all anthracyclines bound to the same binding domain at the inner efflux channel side of P-glycoprotein with high binding affinities. Kaplan–Meier statistics of RNA sequencing data of tumor biopsies of TCGA database revealed that high *ABCB1* expression was significantly correlated with worse survival times for leukemia, multiple myeloma, and hepatocellular carcinoma patients. This indicates that *ABCB1* may serve as a prognostic marker in anthracycline-based chemotherapy regimens in these tumor types and target for the development of novel anthracycline derivatives.

## Figures and Tables

**Table 1:** NSC codes as well as trivial and chemical names of 30 anthracyclines.

Compound No	NSC Code	Trivial Name	Chemical Name
**1a** (3″*S*)	NSC 357704		3′-Deamino-3′-(4″-(3″-cyano)morpholinyl)-doxorubicin
**1b** (3″*R*)	NSC 357704		3′-Deamino-3′-(4″-(3″-cyano)morpholinyl)-doxorubicin
**2a** (13*R*)	NSC 639655		3’-Deamino-3′-(4″-morpholinyl)-13-dihydro-doxorubicin
**2b** (13*S*)	NSC 639655		3′-Deamino-3′-(4″-morpholinyl)-13-dihydro-doxorubicin
**3**	NSC 354646		3′-Deamino-3′-(4″-morpholinyl)-doxorubicin
**4**	NSC 256439	Idarubicin	4-Demethoxydaunorubicin
**5**	NSC 261045		*N*,*N*-Dimethyldoxorubicin
**6**	NSC 623128		14-Fluoro-4-demethoxydaunorubicin
**7**	NSC 208734	Aclarubicin	(1S,2S,4R)-Methyl-4-(((2S,5R,6R)-4-(dimethylamino)-5-(((1S,3R,4S)-3-hydroxy-5-methyl-4-(((2S,6R)-6-methyl-5-oxotetrahydro-2H-pyran-2-yl)oxy)cyclohexyl)oxy)-6-methyltetrahydro-2H-pyran-2-yl)oxy)-2-ethyl-2,5,7-trihydroxy-6,11-dioxo-1,2,3,4,6,11-hexahydrotetracene-1-carboxylate
**8**	NSC 258812		*N*,*N*-Dimethyldaunorubicin
**9**	NSC 83142	Daunorubicin	(7*S*,9*S*)-9-acetyl-7-[(2*R*,4*S*,5*S*,6*S*)-4-amino-5-hydroxy-6-methyloxan-2-yl]oxy-6,9,11-trihydroxy-4-methoxy-8,10-dihydro-7H-tetracene-5,12-dione
**10**	NSC 267469		4′-Deoxydoxorubicin
**11**	NSC 650931		2′-Bromo-4′-*epi*-daunorubicin
**12**	NSC 301739	Mitoxantrone	1,4-dihydroxy-5,8-bis [2-(2-hydroxyethylamino)ethylamino]anthracene-9,10-dione
**13**	NSC 759155	Doxorubicin	14-Hydroxydaunorubicine
**14a** (1″*R*)	NSC 639659		*N*-(1″-Cyano-2″-methoxyethyl)doxorubicin
**14b** (1″*S*)	NSC 639659		*N*-(1″-Cyano-2″-methoxyethyl)doxorubicin
**15**	NSC 149584		Doxorubicin 14-octanoate
**16**	NSC 149583		Doxorubicin 14-nicotinate
**17**	NSC 149585		Doxorubicin 14-benzoate
**18**	NSC 333054	Pirarubicin	4′-*O*-Tetrahydropyranyldoxorubicin
**19**	NSC 759195	Epirubicin	4′-*epi*-Doxorubicin
**20**	NSC 219977	Chlorozorubicin	Chlorobenzoylhydrazone daunorubicin
**21**	NSC 164011	Zorubicin/Rubidazone	Benzoylhydrazone daunorubicin
**22**	NSC 254681		5-Iminodaunorubicin
**23**	NSC 246131		*N*-Trifluoroacetyldoxorubicin-14-valerate
**24**	NSC 143491		Daunorubicin 13-oxime
**25**	NSC 180510		13-Dihydrodaunorubicin
**26**	NSC 284682		3′-Deamino-3′-hydroxydaunorubicin
**27**	NSC 788321		4-(*N*-(2-(4-Fluorophenyl)bicyclo [2.2.1]heptan-2-yl)-*N*-methylamino)-*N*-(doxorubicin)hexanamide
**28**	NSC 378901		4′-Deoxy-4′-iododoxorubicin
**29**	NSC 109351	Daunomycinone	Daunorubicin aglycone
**30**	NSC 268242		*N*,*N*-Dibenzyldaunorubicin

**Table 2:** IC_50_ values (µM) and degrees of resistance of selected anthracyclines as determined by the resazurin assay.

	CCRF-CEM	CEM/ADR5000	Degree of Resistance
Doxorubicin (**13**)	0.042 ± 0.003	41.51 ± 8.17	988.3
Daunorubicin (**9**)	0.012 ± 0.000	9.89 ± 1.37	824.2
Idarubicin (**4**)	0.005 ± 0.000	0.23 ± 0.04	46.0
Aclarubicin (**7**)	0.145 ± 0.015	0.38 ± 0.03	2.6

**Table 3:** Molecular docking of 30 anthracyclines to human P-glycoprotein.

Compound No	Binding Energy (kcal/mol)	Inhibition Constant (pK_i_, nM)	Hydrogen Bonds and Polar Interactions	Hydrophobic and Aromatic Interactions
**1a (3″** ***S*** **)**	−11.9 ± 0.1	2.03 ± 0.20	Tyr 307, Ser 979, Tyr 953	Leu 65, Met 68, Ile 340, Phe 732, Met 949, Tyr 950, Phe 983, Met 986
**1b (3″** ***R*** **)**	−11.6 ± 0.1	3.35 ± 0.76	Tyr 310, Tyr 953, Ser 979	Leu 65, Met 68, Phe 728, Met 949, Tyr 950, Phe 983
**2a (13** ***R*** **)**	−11.3 ± 0.2	5.74 ± 1.38	Gln 725, Phe 983, Gln 990	Met 69, Phe 72, Phe 336, Leu 339, Ile 340, Phe 732, Tyr 953, Phe 978
**2b (13** ***S*** **)**	−11.0 ± 0.1	8.99 ± 1.74	Gln 725, Phe 983, Gly 990	Met 69, Phe 72, Phe 336, Leu 339, Ile 340, Tyr 953
**3**	−12.4 ± 0.0	0.84 ± 0.05	Tyr310, Tyr 953, Leu 975	Leu 65, Met 68, Phe 72, Ile 340, Phe 728, Phe 732, Met 949, Tyr 950, Phe 978, Phe 983
**4**	−10.4 ± 0.1	24.08 ± 3.16	Lys 189, Gln 347	Ala 229, Trp 232, Ala 233, Leu 236, Phe 343, Pro 350, Met 876
**5**	−11.7 ± 0.0	2.90 ± 0.07	Tyr 310, Tyr 953	Leu 65, Met 68, Ile 340, Phe 728, Met 949, Tyr 950, Phe 983
**6**	−10.4 ± 0.2	25.11 ± 6.45	Tyr 310, Gln 990	Tyr 307, Phe 336, Phe 732, Phe 978, Phe 983, Ala 987
**7**	−15.1 ± 0.2	(9.41 ± 3.34) × 10^−3^	Gln 195, Ile 340, Ser 344, Gln 725, Tyr 953, Ser 979	Leu 65, Met 69, Phe 336, Phe 983
**8**	−11.0 ± 0.1	8.74 ± 1.87	Ala 229, Trp 232, Gln 990	Leu 236, Ile 299, Phe 303, Phe 770, Met 876, Leu 879, Phe 994
**9**	−10.9 ± 0.1	9.96 ± 1.32	Tyr 310, Leu 339, Ile 340, Gln 347, Gln 725, Glu 875, Phe 983	Met 986, Ala 987
**10**	−11.4 ± 0.4	5.55 ± 3.08	Tyr 310, Tyr 953	Leu 65, Met 68, Phe 72, Phe 336, Phe 732, Met 949, Tyr 950, Phe 978, Phe 983
**11**	−11.0 ± 0.0	8.98 ± 0.28	Tyr 310, Gln 725, Tyr 953, Ser 979	Leu 65, Met 68, Ile 340, Phe 728, Met 949, Tyr 950
**12**	−10.3 ± 0.2	29.1 ± 9.20	Phe 983, Tyr 950, Tyr 953	Phe 336
**13**	−12.2 ± 0.0	1.16 ± 0.08	Lys 189, Ser 237, Gln 347, Met 876, Ser 993	Ala 229, Trp 232, Ala 233, Leu 236, Phe 343, Phe 994
**14a (1″** ***R*** **)**	−11.9 ± 0.1	1.88 ± 0.36	Tyr 310, Gln 725, Leu 975	Tyr 307, Phe 336, Phe 728, Phe 732, Phe 978, Phe 983
**14b (1″** ***S*** **)**	−11.6 ± 0.1	3.07 ± 0.53	Trp 232, Gln 838	Ile 235, Leu 236, Phe 239, Ala 295, Ile 299, Met 876
**15**	−15.4 ± 0.4	(6.23 ± 5.00) × 10^−3^	Asn 721, Gln 725, Gln 838, Gln 990	Trp 232, Phe 303, Phe 336, Phe 728, Phe 732, Met 876, Phe 983, Val 991, Phe 994
**16**	−14.7 ± 0.1	(17.37 ± 3.00) × 10^−3^	Tyr 307, Asn 721, Gln 725, Gln 838, Ser 979	Trp 232, Phe 303, Phe 728, Phe 983, Phe 994
**17**	−14.6 ± 0.1	(20.01 ± 4.24) × 10^−3^	Tyr 310, Gln 725, Glu 875, Met 986	Leu 65, Phe 343, Phe 728, Phe 732, Met 949, Tyr 950, Tyr 953, Phe 983
**18**	−13.1 ± 0.0	0.25 ± 0.02	Tyr 307, Gln 725, Tyr 953	Met 69, Phe 72, Ile 306, Phe 336, Leu 339, Ile 340, Phe 343, Phe 983
**19**	−11.8 ± 0.1	2.38 ± 0.43	Lys 189, Phe 239, Thr 240, Ser 880, Ser 993	Ala 233, Leu 236, Pro 350, Leu 879, Phe 994
**20**	−15.1 ± 0.1	(9.22 ± 1.6) × 10^−3^	Tyr 310, Asn 721, Gln 990	Ile 299, Trp 232, Phe 303, Gln 725, Phe 732, Phe 770, Met 876, Leu 975, Phe 978, Phe 983, Ala 987
**21**	−14.4 ± 0.0	(26.6 ± 1.5) × 10^−3^	Trp 232, Glu 875, Gln 990	Ala 233, Phe 303, Phe 343, Pro 350, Met 876, Leu 879, Ala 987, Val 991
**22**	−10.8 ± 0.0	11.17 ± 0.17	Glu 875, Gln 990	Trp 232, Phe 303, Phe 343, Met 876, Ala 987, Val 991
**23**	−16.2 ± 0.6	(1.86 ± 1.41) × 10^−3^	Tyr 307, Gln 725, Tyr 953, Gln 990	Met 69, Phe 72, Phe 336, Leu 339, Ile 340, Phe 343, Phe 983, Ala 987
**24**	−11.0 ± 0.2	9.59 ± 2.83	Lys 189, Ala 233, Leu 236, Met 876, Leu 879	Trp 232, Phe 343, Ser 880, Lys 877
**25**	−11.2 ± 0.1	6.79 ± 1.34	Gln 347, Met 876	Trp 232, Ala 233, Leu 236, Phe 343, Pro 350, Lys 877, Leu 879, Ser 880
**26**	−11.3 ± 0.1	5.02 ± 0.96	Tyr 307, Tyr 310, Ser 979	Met 69, Phe 72, Phe 336, Leu 339, Ile 340, Phe 983
**27**	−17.5 ± 0.5	(0.18 ± 0.13) × 10^−3^	Tyr 310, Gln 725, Leu879, Leu 975, Gln 990	Trp 232, Ala 233, Leu 236, Tyr 307, Phe 336, Phe 343, Phe 372, Phe 728, Phe 983
**28**	−11.5 ± 0.0	3.87 ± 0.19	Trp 232, Glu 875, Gln 990	Phe 303, Phe 343, Met 876, Ala 987, Val 991
**29**	−9.5 ± 0.0	103.74 ± 7.08	Lys 189, Ser 237, Gln 347	Ala 233, Leu 236, Met 876, Leu 879, Ser 880, Phe 994
**30**	−14.9 ± 0.0	(12.15 ± 0.9) × 10^−3^	Tyr 953, Ser 979	Leu 65, Met 68, Tyr 307, Phe 732, Met 949, Tyr 950, Phe 983, Met 986, Ala 987
**Elacridar**	−14.3 ± 0.1	(35.49 ± 3.5) × 10^−3^	Lys 189	Trp 232, Ala 233, Leu 236, Phe 303, Pro 350, Phe 770, Met 876, Leu 879, Val 991, Phe 994

## Data Availability

The original contributions presented in this study are included in the article/Supplementary Materials. Further inquiries can be directed to the corresponding author.

## Supplementary Materials

The following supporting information can be downloaded at https://www.mdpi.com/article/10.3390/biom15070971/s1, Figure S1: Representative merged images of fluorescent and phase-contrast channels of CCRF-CEM and CEM/ADR5000 cells treated with the indicated compounds; Table S1: Log10IC50 values of the NCI cell line panel for anthracyclines.

## Abbreviations

The following abbreviations are used in this manuscript:

ABC	ATP-binding cassette
KM	Kaplan–Meier
miR	Micro-RNA
NCI	National Cancer Institute
TCGA	The Cancer Genome Atlas
qRT-PCR	Real-Time Quantitative Reverse Transcription PCR
