# Supplementary material for: Predictive and Prognostic Relevance of ABC Transporters for Resistance to Anthracycline Derivatives
Source: Biomolecules. 2025 Jul 6;15(7):971. doi: 10.3390/biom15070971 (PMC12292137; doi:10.3390/biom15070971)
Supplement: Supplementary file 1 [file biomolecules-15-00971-s001.zip › biomolecules-3469935-supplementary.pdf]

Supplementary Materials

Table S1: $Log_{10}IC_{50}$ values of the NCI cell line panel for anthracyclines.

	compound A	compound B	compound C	compound 30	compound 29 (Daunomycinone)	compound 28	compound 27	compound 26	compound 25	compound 24	compound 23 (valrubicin)	compound 22	compound 21 (zorubicin)	compound 20 (chlorozorubicin)	compound 19 (epirubicin)
**Cell line (n=58)**	**169534**	**307241**	**722333**	**268242**	**109351**	**378901**	**788321**	**284682**	**180510**	**143491**	**246131**	**254681**	**164011**	**219977**	**759195**
CCRF-CEM	-0.096	0.379		-5.615	-5.726	-5.966	-5.947	-7.045	-6.831	-6.728	-6.869	-7.486	-7.228	-7.044	-7.037
HL-60(TB)	0.454	0.905	-2.138	-5.020	-5.444	-6.369	-5.181	-7.300	-6.766	-6.662	-6.901	-7.572	-7.219	-6.877	-6.838
K-562	0.973	0.491	-1.108	-5.439	-5.363	-5.322	-5.955	-6.034	-5.952	-6.257	-6.472	-6.484	-6.633	-6.634	-6.832
MOLT-4	0.051	-0.011		-5.825	-5.703	-6.475	-6.517	-7.681	-6.978	-7.180	-7.642	-8.061	-7.489	-7.264	-7.486
RPMI-8226	0.860	0.315		-5.335	-5.661	-5.347	-6.046	-6.314	-6.540	-6.228	-6.742	-6.915	-6.863	-6.666	-6.793
SR	-0.333	0.181		-6.025			-6.652	-7.052	-6.796	-7.211	-7.518	-7.709	-7.702	-7.414	-7.864
A549/ATCC	0.620	0.454	-2.112	-5.348	-5.184	-6.133	-6.217	-6.333	-6.025	-6.014	-6.713	-7.022	-6.687	-6.906	-7.072
EKVX	1.875	0.652	-1.410	-4.404	-5.310	-4.841	-5.105	-5.176	-5.927	-5.496	-5.614	-6.054	-6.159		-5.919
HOP-62	0.766	0.492	-2.098	-4.582	-5.670	-5.796	-5.693	-6.513	-6.065	-5.907	-6.737	-6.902	-6.915	-6.715	-6.796
HOP-92	1.379	1.468	-1.268	-4.750	-5.148	-5.014	-5.271	-5.678	-6.299	-5.966	-6.425	-6.482	-6.943	-6.718	-7.129
NCI-H226	0.243	0.762	-2.080	-4.882	-5.255	-6.104	-5.176	-6.539	-6.217	-5.742	-7.397	-6.831	-6.974	-6.825	-7.168
NCI-H23	1.052	0.134	-2.201	-4.648	-5.312	-5.643	-5.457	-6.026	-6.257	-5.898	-6.455	-6.805	-6.697	-6.627	-6.912
NCI-H322M	1.653	0.622	-1.467	-4.764	-5.234	-4.825	-5.132	-5.346	-5.318	-5.435	-5.644	-5.754	-5.989	-6.152	-5.847
NCI-H460	-0.149	-0.112	-2.201	-6.186	-5.807	-6.505	-6.256	-6.906	-6.483	-6.572	-7.670	-7.735	-7.426	-7.330	-7.636
NCI-H522	0.610	0.309	-1.975	-4.930	-5.989	-5.537	-6.151	-5.740	-6.244	-6.502	-6.133	-6.305	-6.722	-6.905	-6.997
COLO205	1.000	0.391	-1.615	-4.745	-5.520	-4.877	-6.138	-5.689	-5.851	-6.020	-5.956	-6.886	-6.324	-6.692	-6.504
HCC-2998	1.571	0.809	-1.716	-4.340	-4.897	-4.742	-5.986	-6.037	-5.776	-6.079	-5.886	-6.331	-6.318	-6.393	-6.794
HCT-116	0.861	0.159	-2.129	-5.736	-5.618	-6.242	-6.137	-6.541	-6.094	-6.055	-6.531	-6.889	-6.710	-6.935	-6.952
HCT-15	1.875	1.197	-1.033	-5.308	-5.035	-5.737	-5.659	-5.846	-5.082	-4.542	-5.858	-5.767	-5.741	-5.991	-5.524
HT29	1.195	0.416	-1.693	-5.024	-5.219	-5.502	-6.170	-5.851	-5.656	-5.874	-6.131	-6.369	-6.230	-6.635	-6.527
KM12	1.228	0.774	-1.672	-4.815	-5.210	-4.519		-5.811	-5.649	-5.621	-6.104	-6.428	-6.420	-6.486	-6.299
SW-620	0.854	0.450	-1.950	-5.329	-5.632	-5.511	-6.119	-6.271	-5.977	-6.298	-6.583	-6.565	-6.873	-6.991	-7.057
SF-268	0.875	0.694	-2.201	-4.772	-5.374	-4.798	-5.269	-5.967	-5.980	-6.031	-6.396	-6.577	-6.552	-6.656	-6.707
SF-295	1.270	0.658	-2.201	-4.693	-5.242	-5.677	-5.237	-6.172	-5.901	-5.860	-6.512	-6.661	-6.722	-6.628	-6.802
SF-539	1.583	0.398	-1.767	-4.964	-5.325	-5.581	-5.435	-6.046	-6.061	-6.031	-6.412	-6.807	-6.584	-6.712	-6.872
SNB-19	0.309	0.567	-2.201	-4.663	-5.220	-5.506	-5.188	-5.961	-6.054	-6.108	-6.458	-6.498	-6.752	-6.790	-6.895
SNB-75	0.960	0.227	-2.201	-4.410	-5.014	-5.599	-5.484	-5.791	-5.767	-6.300	-5.992	-6.595	-6.428	-6.990	-7.076
U251	0.346	0.222	-2.201	-4.813	-5.395	-5.569	-6.131	-6.445	-6.053	-6.116	-6.655	-6.807	-6.833	-6.954	-7.063
LOXIMVI	0.590		-2.201	-5.301	-5.788	-5.840	-5.286	-6.229	-6.281	-6.411	-6.787	-6.773	-7.131	-7.121	-7.083
MALME-3M	1.000	0.966	-1.943	-4.814	-5.341	-5.218	-5.105	-5.852	-6.309	-6.122	-6.148	-6.563	-6.671	-7.119	-6.837
M14	0.971	0.470	-2.195	-4.445			-5.458	-5.568	-5.520	-5.643	-6.080	-6.152	-6.693	-6.532	-6.551

	compound 18	compound 17	compound 16	compound 15	compound 14	compound 13 (doxorubicin)	compound 12 (mitoxantrone)	compound 11	compound 10	compound 9 (daunorubicin)	compound 8	compound 7 (aclarubicin)	compound 6	compound 5	compound 4 (idarubicin)	compound 3	compound 2	compound 1
**Cell line (n=58)**	**333054**	**149585**	**149583**	**149584**	**639659**	**759155**	**301739**	**650931**	**267469**	**83142**	**258812**	**208734**	**623128**	**261045**	**256439**	**354646**	**639655**	**357704**
CCRF-CEM	-7.426	-7.238	-7.241	-7.611	-7.695	-7.731	-8.223	-8.383	-7.574	-8.000	-7.536	-7.640	-8.000	-7.506	-8.000	-7.645	-8.071	-10.547
HL-60(TB)	-8.000	-7.118	-6.834	-7.580	-7.715	-7.108	-7.986	-8.013	-7.542	-7.942	-7.395	-7.637	-8.000	-8.000	-8.000	-7.720	-8.227	-10.606
K-562	-6.725	-6.946	-6.824	-7.254	-7.382	-7.051	-6.928	-7.305	-7.490	-7.827	-8.341	-7.648	-7.816	-7.851	-7.963	-7.695	-8.385	-10.302
MOLT-4	-8.000	-7.519	-7.433	-7.943	-7.865	-7.732	-8.323	-9.000	-7.459	-8.000	-8.305	-7.632	-8.000	-7.799	-8.000	-8.133	-8.507	-10.497
RPMI-8226	-6.952	-6.768	-6.943		-7.550	-7.041	-6.730	-7.807	-7.264	-7.866	-7.946	-7.615	-8.000	-7.758	-7.883	-8.119	-8.422	-10.412
SR		-7.888	-7.761	-8.000	-7.953	-8.253	-8.117	-8.304	-7.699	-7.869	-8.625	-7.648	-8.000	-8.000	-8.000	-8.332	-8.670	-10.896
A549/ATCC	-8.000	-7.073	-7.137	-7.385	-7.283	-7.535	-7.942	-7.695	-7.617	-7.647	-7.093	-7.648	-8.000	-8.000	-8.000	-8.133	-8.966	-10.481
EKVX	-6.038			-6.650	-6.301	-6.696	-6.539	-6.094	-7.072	-6.576	-6.901	-7.382	-6.295	-7.382	-7.083	-7.383	-8.413	-9.553
HOP-62	-6.673	-6.790	-6.906	-7.131	-7.542	-7.481	-7.894	-7.833	-7.655	-7.896	-6.933	-7.499	-8.000	-7.565	-7.901	-7.702	-8.501	-10.537
HOP-92	-6.622	-7.195	-7.066		-6.406	-7.628	-7.752	-7.313	-7.381	-7.981	-7.076	-7.464	-7.316	-7.509	-7.783	-7.721	-7.187	-10.213
NCI-H226	-7.094	-6.954	-6.959	-7.407	-7.556	-7.696	-7.814	-7.782	-7.474	-7.620	-7.538	-7.524	-8.000	-7.826	-7.965	-8.250	-9.000	-10.768
NCI-H23	-7.292	-6.619	-6.750	-7.258	-7.228	-7.315	-7.178	-7.569	-7.499	-7.629	-7.405	-7.642	-8.000	-7.863	-7.922	-7.665	-8.334	-10.362
NCI-H322M	-6.178	-6.476	-6.437	-6.391	-6.560	-6.544	-6.587	-6.637	-7.385	-7.014	-7.555	-7.648	-6.594	-7.797	-7.105	-7.896	-8.571	-10.312
NCI-H460	-8.000	-7.748	-7.570	-8.000	-8.000	-8.252	-8.249	-9.000	-7.593	-8.000	-8.105	-7.648	-8.000	-8.000	-8.000	-8.520	-9.000	-10.962
NCI-H522	-6.448	-6.988	-7.241	-7.647	-7.493	-7.477	-7.264	-7.186	-7.420	-7.810	-7.444	-7.648	-7.694	-7.959	-7.867	-7.739	-8.563	-10.221
COLO205	-6.483	-6.779	-6.774	-7.090	-7.316	-7.044	-6.968	-6.379	-7.377	-6.806	-7.875	-7.648	-7.529	-8.000	-7.645	-7.980	-8.753	-9.871
HCC-2998	-6.207	-6.376	-6.343	-6.863	-6.838	-7.169	-6.623	-6.954	-7.114	-6.813	-7.478	-7.335	-6.789	-7.171	-7.704	-7.895	-9.000	-10.246
HCT-116	-7.182	-7.176	-7.246	-7.297	-7.414	-7.491	-7.257	-8.101	-7.636	-7.869	-8.647	-7.648	-8.000	-8.000	-8.000	-8.586	-9.000	-10.007
HCT-15	-6.328	-6.336	-5.969	-6.153	-6.096	-6.043	-6.627	-6.882	-6.994	-6.142	-7.676	-7.602	-7.232	-7.740	-7.811	-7.826	-8.452	-10.293
HT29	-6.640	-6.991	-6.700	-7.161	-6.821	-7.019	-6.603	-6.818	-7.456	-7.609	-8.287	-7.644	-7.806	-8.000	-7.932	-8.202	-8.512	-10.360
KM12	-6.426	-6.667	-6.546	-6.726	-6.988	-6.868	-6.338	-6.855	-7.360	-7.354	-7.766	-7.613	-7.516	-7.435	-7.777	-7.611	-8.259	-10.197
SW-620	-7.224	-7.349	-7.218	-7.461	-7.481	-7.506	-7.266	-7.752	-7.515	-7.777	-8.315	-7.648	-7.937	-8.000	-7.942	-8.027	-8.842	-10.302
SF-268	-6.533	-6.798	-6.810	-7.267	-7.378	-7.210	-7.541	-6.996	-7.346	-7.737	-7.720	-7.163	-7.594	-7.901	-7.899	-7.621	-8.637	-10.445
SF-295	-7.316	-6.994	-6.831	-7.315	-7.015	-7.431	-7.675	-7.355	-7.621	-7.833	-7.725	-7.534	-8.000	-8.000	-8.000	-7.843	-8.686	-10.583
SF-539	-6.909	-6.657	-7.128	-6.976	-7.382	-7.412	-7.750	-7.234	-7.423	-7.408	-7.762	-7.593	-7.251	-8.000	-7.937	-7.812	-8.366	-10.465
SNB-19	-7.023	-6.741	-7.161	-7.291	-7.370	-7.609	-7.811	-7.385	-7.489	-7.869	-7.402	-7.493	-7.854	-7.595	-7.914	-7.693	-8.394	-10.285
SNB-75	-6.616	-6.984	-7.076	-7.225	-7.249	-7.469	-7.850	-8.537	-7.396	-7.794	-6.860	-7.546	-7.792	-7.810	-7.864	-8.083	-8.411	-10.542
U251	-7.182	-6.901	-7.177	-7.384	-7.544	-7.603	-8.041	-7.530	-7.560	-7.839	-7.345	-7.608	-7.886	-7.872	-7.955	-7.968	-8.328	-10.503
LOXIMVI	-6.635	-7.333	-7.354	-7.403	-7.534	-7.597	-7.686	-7.681	-7.582	-7.884	-8.413	-7.648	-8.000	-8.000	-7.980	-8.333	-8.772	-10.637
MALME-3M	-7.125	-6.835	-6.940	-7.365	-7.464	-7.336	-6.921	-7.114	-7.546	-7.519	-7.392	-7.610	-7.963	-7.847	-8.000	-7.847	-8.600	-9.984
M14	-6.558	-6.574	-6.769	-7.063	-6.874	-7.073	-6.927	-7.167	-7.444	-7.313	-7.360	-7.648	-7.580	-7.514	-7.838	-7.490	-8.530	-10.266

Table S1 (Continued): $Log_{10}IC_{50}$ values of the NCI cell line panel for anthracyclines.

	compound A	compound B	compound C	compound 30	compound 29 (Daunomycinone)	compound 28	compound 27	compound 26	compound 25	compound 24	compound 23 (valrubicin)	compound 22	compound 21 (zorubicin)	compound 20 (chlorozorubicin)	compound 19 (epirubicin)	compound 18	compound 17	compound 16	compound 15	compound 14	compound 13 (doxorubicin)	compound 12 (mitoxantrone)	compound 11	compound 10	compound 9 (daunorubicin)	compound 8	compound 7 (aclarubicin)	compound 6	compound 5	compound 4 (idarubicin)	compound 3	compound 2	compound 1
**Cell line (n=58)**	**169534**	**307241**	**722333**	**268242**	**109351**	**378901**	**788321**	**284682**	**180510**	**143491**	**246131**	**254681**	**164011**	**219977**	**759195**	**333054**	**149585**	**149583**	**149584**	**639659**	**759155**	**301739**	**650931**	**267469**	**83142**	**258812**	**208734**	**623128**	**261045**	**256439**	**354646**	**639655**	**357704**
MDA-MB-435				-4.635	-5.237	-5.569		-5.796	-5.936	-6.129	-6.480		-6.520							-7.277		-7.039		-7.503	-7.057	-7.671	-7.648	-7.750	-8.000	-8.000	-7.971	-9.000	-9.872
SK-MEL-2	1.381	1.049	-1.658	-4.687	-5.093	-5.009	-5.928	-5.318	-5.852	-5.957	-6.017		-6.297	-6.584	-6.289	-5.979	-6.250	-6.635	-7.157	-6.866	-6.850	-6.410	-6.574	-7.194	-6.915	-7.098	-7.517	-6.774	-7.673	-8.000	-7.689	-8.375	-10.287
SK-MEL-28	1.528	1.027	-1.837	-4.368	-4.897	-4.817	-5.140	-5.216	-5.849	-5.786	-5.761	-5.799	-6.003	-6.497	-6.455	-5.854	-6.309	-6.692	-6.840	-6.807	-7.099	-6.330	-6.498	-7.087	-6.776	-7.324	-7.333	-6.753	-7.645	-7.310	-7.417	-8.391	-9.714
SK-MEL-5	0.677	0.952	-1.887	-4.770	-5.665	-5.579	-5.117	-6.058	-6.079	-6.192	-6.485	-6.530	-6.794	-6.867	-6.959	-6.939	-6.721	-6.930	-7.370	-7.518	-7.434	-7.258	-7.162	-7.430	-7.745	-7.434	-7.550	-7.947	-7.891	-7.923	-7.700	-8.651	-10.534
UACC-257	1.476	1.068	-0.990	-4.434	-4.974	-4.533	-6.031	-5.422	-5.849	-5.750	-5.886	-5.969	-6.207	-6.740	-6.365	-6.002	-6.581	-6.764	-7.293	-7.041	-6.992	-5.724	-6.811	-7.292	-7.164	-7.362	-7.616	-7.100	-7.545	-7.424	-7.725	-8.471	-10.236
UACC-62	0.877	0.331	-2.201	-4.779	-5.253	-5.800	-5.183	-5.981	-5.874	-5.950	-6.734	-6.513	-6.616	-6.784	-7.072	-6.681	-6.762	-6.986	-7.333	-7.241	-7.461	-7.413	-6.978	-7.596	-7.780	-7.429	-7.640	-7.984	-7.921	-7.911	-8.238	-9.000	-10.455
IGROV1	1.207	0.835	-1.678	-5.020	-5.463	-5.726	-5.181	-5.516	-5.658	-6.119	-6.100	-5.955	-6.581	-6.859	-6.756	-6.373	-7.106	-6.919	-7.949	-7.394	-7.045	-6.733	-6.575	-7.421	-7.428	-7.662	-7.648	-7.057	-7.981	-7.417	-7.883	-8.554	-9.716
OVCAR-3	1.354	0.765	-1.960	-4.533	-5.108	-4.476	-5.850	-5.433	-5.619	-5.838	-5.528	-5.972	-6.194	-6.403	-6.213	-6.082	-6.433	-6.542	-6.942	-6.816	-6.777	-6.545	-6.867	-7.262	-7.178	-6.968	-7.606	-6.632	-7.191	-7.426	-7.566	-8.638	-10.099
OVCAR-4	1.669	0.623	-1.814	-4.741	-5.198	-5.397	-5.961	-5.419	-5.302	-5.351	-5.681	-5.785	-5.931	-6.458	-6.263	-6.181	-6.711	-6.554	-6.814	-6.550	-6.876	-6.480	-6.732	-7.337	-7.192	-7.515	-7.644	-6.817	-7.723	-7.179	-7.697	-8.473	-10.137
OVCAR-5	1.875	0.648	-1.638	-4.601	-4.912	-4.870	-6.005	-5.982	-5.482	-5.568	-5.818	-6.122	-6.192	-6.422	-5.895	-6.534	-6.398	-6.476	-6.375	-6.607	-6.669	-6.512	-6.553	-7.131	-6.697	-7.576	-7.496	-7.143	-7.192	-7.583	-7.718	-8.323	-10.238
OVCAR-8	1.289	0.526	-1.988	-5.311	-5.546	-5.446	-5.928	-5.887	-5.942	-5.940	-6.285	-6.525	-6.549	-6.674	-6.592	-6.573	-6.880	-6.739	-6.880	-7.170	-7.174	-7.435	-7.254	-7.467	-7.697	-8.143	-7.648	-7.938	-7.594	-7.832	-8.177	-8.741	-10.298
SK-OV-3	1.354	0.563	0.050	-4.331	-4.940	-5.400	-5.127	-5.346	-5.566	-5.651	-6.463	-6.530	-6.235	-6.318	-6.497	-6.276	-6.578	-6.418	-7.118	-7.061	-6.970	-7.367	-7.347	-7.283	-7.724	-6.842	-7.355	-7.712	-7.138	-7.891	-7.419	-8.442	-9.816
786-0	0.815	0.300	-2.201	-5.497			-6.086	-5.795	-6.191	-5.974	-6.635	-6.766	-7.250	-6.604	-6.911		-6.760	-6.853	-7.304	-7.364	-7.489	-7.667	-7.394	-7.699	-7.750	-7.250	-7.648	-8.000	-7.705	-7.945	-7.946	-9.000	-10.272
A498	1.280	0.771	-1.663	-4.419	-5.998	-5.453	-5.228	-5.516	-5.661	-6.079	-6.608	-6.014	-6.292	-6.890	-6.875	-7.386	-7.113	-6.946	-6.810	-7.417	-7.317	-7.208	-6.875	-7.505	-6.706	-6.859	-7.602	-6.633	-7.937	-7.885	-7.955	-8.869	-10.476
ACHN	1.306	0.481	-2.201	-5.567			-5.757	-6.237	-5.822	-5.721	-6.393	-6.612	-7.096	-6.743	-6.705		-7.069	-6.932	-7.232	-7.236	-7.552	-7.941	-7.671	-7.699	-7.959	-8.045	-7.648	-8.000	-8.000	-8.000	-8.290	-8.715	-10.486
CAKI-1	1.213	0.486	-2.201	-5.220	-5.336	-5.542	-5.166	-6.103	-6.390	-5.714	-6.693	-6.852	-6.886	-6.150	-5.881	-7.452	-6.154	-6.152	-6.571	-7.411	-6.745	-8.046		-7.633	-7.087	-8.044	-7.633	-8.000	-8.000	-8.000	-8.023	-8.757	-10.528
RXF393	1.124	0.312	-2.201	-4.402	-5.223	-5.612	-5.566	-5.931	-5.832	-5.851	-6.086	-6.006	-6.481	-6.633	-6.689	-6.367	-6.769	-6.773	-7.039	-6.751	-7.124	-7.186	-6.672	-7.301	-7.221	-7.313	-7.340	-7.196	-7.860	-7.780	-7.686	-9.000	-10.455
SN12C	1.159	0.185	-2.201	-5.535	-5.420	-5.623	-5.240	-6.171	-6.003	-6.130	-6.537	-6.666	-6.811	-6.808	-6.899	-6.831	-7.029	-7.081	-7.438	-7.375	-7.597	-8.106	-7.551	-7.525	-7.746	-7.785	-7.648	-8.000	-7.723	-7.931	-7.806	-8.688	-10.514
TK-10	1.875	1.283	-1.498	-4.746			-6.214	-5.234	-5.602	-5.489	-5.608	-5.882	-6.276	-6.751	-6.138		-6.820	-6.828	-6.773	-6.722	-6.752	-6.374	-6.802	-6.831	-6.842	-6.915	-7.648	-6.999	-7.518	-7.743	-7.314	-8.424	-9.826
UO-31	1.771	0.256	-1.750		-5.019	-5.312	-5.215	-5.779	-5.000	-4.999	-5.973	-5.391	-5.753	-6.312	-6.063	-5.829	-6.354	-6.338	-6.208	-6.355	-6.819	-6.523	-7.021	-6.536	-6.385	-7.237	-7.648	-7.407	-7.691	-7.935	-7.750	-8.700	-10.312
PC-3	1.571	0.996	-1.291	-5.140			-6.003	-5.305		-5.866	-5.945		-6.768	-6.514	-6.259		-6.641	-6.653	-6.717	-7.026	-6.951	-6.850	-6.861	-7.569	-7.319		-7.648	-7.561	-8.000	-7.359	-7.481	-7.911	-12.000
DU-145	1.202	0.383	-2.036	-4.700			-5.948	-5.790		-5.765	-6.439		-6.871	-6.566	-6.798		-6.599	-6.652	-7.575	-7.385	-7.165	-7.472	-7.323	-7.573	-7.484		-7.648	-8.000	-7.285	-7.983	-7.304	-8.153	-12.000
MCF7	0.441	0.505	-2.201	-4.843	-5.995	-5.933	-6.015	-6.815		-6.539			-7.332	-7.389	-7.607	-7.507	-7.520	-7.481	-8.000	-8.000	-8.033	-8.371	-8.415	-7.621	-8.000		-7.648	-8.000		-8.000	-7.552	-7.652	-12.000
MDA-MB-231/ATCC	1.645	1.250	-1.573	-4.850			-5.998	-5.063		-5.601	-5.523		-6.310	-6.251	-6.226		-6.425	-6.486	-6.273	-6.950	-6.842	-6.696	-6.632	-7.213	-7.273		-7.624	-7.367	-7.277	-7.023	-7.259	-7.783	
HS578T	1.529	1.110	-1.022	-4.771			-5.083	-6.001		-5.752	-5.763		-6.592	-6.433	-6.590		-6.460	-6.756	-6.987	-6.792	-6.878	-7.032		-7.117	-6.717		-7.648	-7.986	-8.000	-7.849	-7.604	-7.836	-12.000
BT-549	1.539	0.959	-1.050	-4.561			-5.276	-5.477		-5.764	-5.675		-6.517	-6.299	-6.894		-6.155	-6.715	-6.379	-6.860	-7.229	-7.153	-6.571	-7.548	-7.317		-7.648	-6.883	-7.270	-7.220	-7.281	-7.532	-12.000
T-47D	0.877	0.570	-2.201	-4.799				-5.550		-6.141	-6.268		-6.799	-6.787	-6.893		-6.800	-7.008	-7.379		-7.389	-7.202	-7.233	-7.633	-7.755		-7.607	-7.974	-8.000	-7.840	-7.241		-12.000
**Mean value**	1.061	0.601	-1.809	-4.933	-5.360	-5.466	-5.669	-5.964	-5.965	-5.975	-6.330	-6.544	-6.634	-6.704	-6.713	-6.780	-6.825	-6.865	-7.146	-7.180	-7.239	-7.250	-7.292	-7.414		-7.582	-7.584	-7.587	-7.745	-7.794	-7.813	-8.499	-10.490
**SD**	0.534	0.344	0.456	0.443	0.296	0.505	0.444	0.545	0.413	0.440	0.507	0.554	0.420	0.304	0.461	0.584	0.376	0.343	0.454	0.431	0.410	0.629	0.643	0.224		0.474	0.106	0.491	0.266	0.271	0.316	0.385	0.591

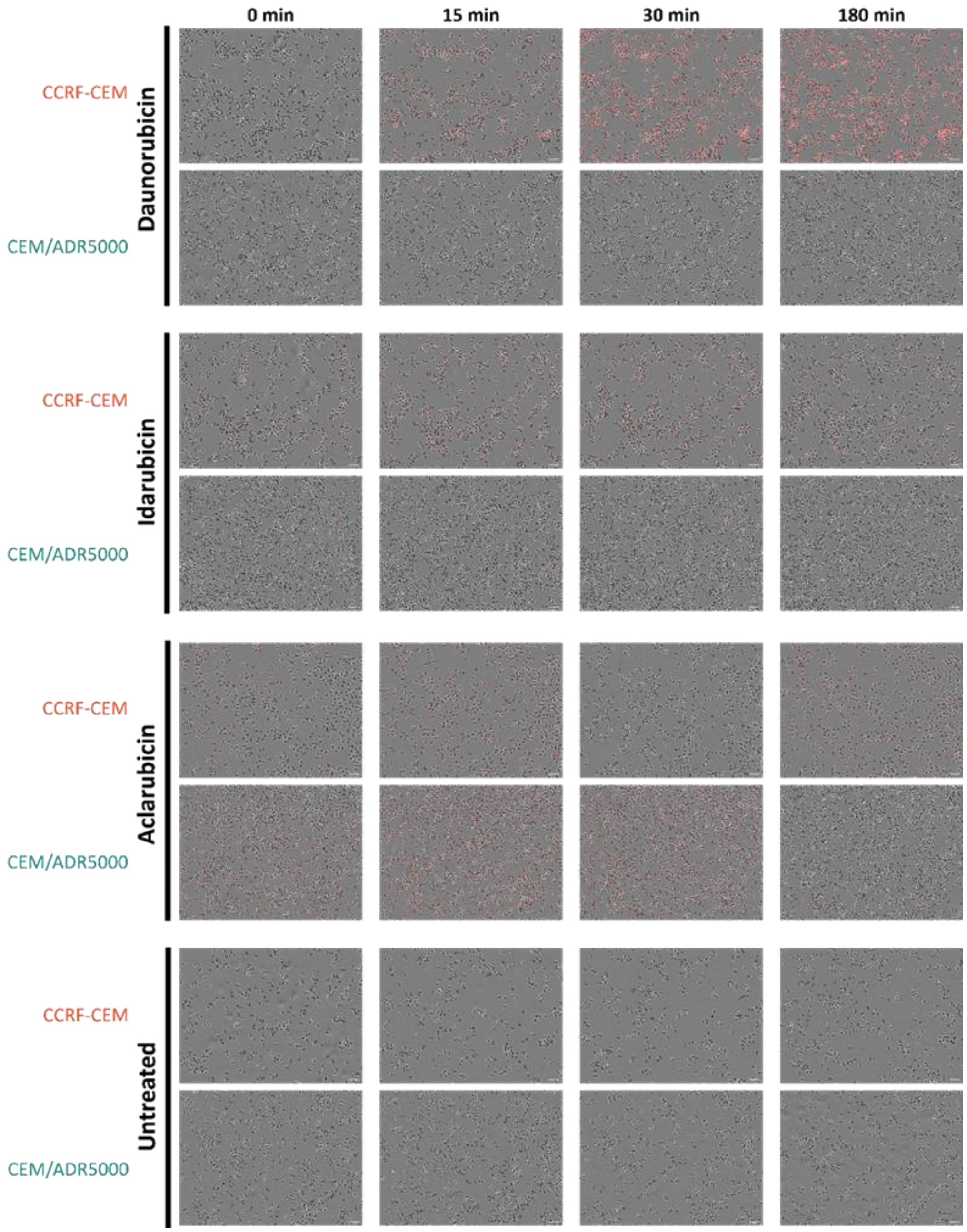

Figure S1. Representative merged images of fluorescent and phase-contrast channels of CCRF-CEM and CEM/ADR5000 cells treated with the indicated compounds (from top to bottom: daunorubicin (**9**), idarubicin (**4**), aclarubicin (**7**), and untreated control), shown at 0, 15, 30, and 180 minutes. For each treatment, images of CCRF-CEM cells are displayed in the first row and CEM/ADR5000 cells in the second row. The scale bar in the merged images corresponds to 50 µm.
